# A responsive living material prepared by diffusion reveals extracellular enzyme activity of cyanobacteria

**DOI:** 10.1073/pnas.2424405122

**Published:** 2025-05-01

**Authors:** Lisa Tang, Nathan T. Soulier, Rebecca Wheeler, Jonathan K. Pokorski, James W. Golden, Susan S. Golden, Jinhye Bae

**Affiliations:** ^a^Aiiso Yufeng Li Family Department of Chemical and Nano Engineering, University of California, San Diego, La Jolla, CA 92093; ^b^Department of Molecular Biology, University of California, San Diego, La Jolla, CA 92093; ^c^Department of Chemical Engineering, Chung-Ang University, Seoul 06794, Republic of Korea

**Keywords:** engineered living materials, biomaterials, cyanobacteria, amidase

## Abstract

Stimuli-responsive engineered living materials (ELMs) can respond to environmental or biochemical cues and have broad utility in biological sensors and machines, but have traditionally been limited to biocompatible scaffolds. This is because they are typically made by mixing cells into a precursor solution before crosslinking. Here, we demonstrate a diffusion mechanism for incorporating cells of the cyanobacterium *Synechococcus elongatus* sp. PCC 7942 (*S. elongatus*) into nanoclay-poly-N-isopropylacrylamide (NC-PNIPAm), a hydrogel with a cytotoxic precursor, by exploiting its temperature-dependent shape-morphing behavior. Subsequent growth of *S. elongatus* caused a decrease in the bending curvature and stiffness (local Young’s modulus) of NC-PNIPAm due to partial degradation by an unannotated enzyme. Creation and observation of this cyanobacteria-hydrogel ELM showcases a method for diffusing cells into a hydrogel as well as characterizing an extracellular enzyme.

## Significance

Previously described engineered living materials (ELMs) have been created with nonresponsive, biocompatible polymers by adding living cells to a precursor solution before polymerization, which cells must survive. We diffused photosynthetic cyanobacteria into a temperature-responsive polymer postpolymerization, circumventing its toxic precursor to create an ELM responsive to temperature, with the potential to add responsive behaviors based on innate or engineered capabilities of cyanobacteria. This stimuli-responsive ELM underwent an irreversible shape change as the cells grew. This change was attributed to a decrease in polymer stiffness, caused by a previously undescribed enzyme secreted by some cyanobacteria. The creation and study of this ELM demonstrates the utilization of materials with toxic precursors, benefits of observing materials in nonequilibrium states, and cyanobacteria–polymer matrix interactions.

Stimuli-responsive composite hydrogels have been studied for application in sensors (1), actuators (2, 3), and soft robotics (4) because of their ability to undergo programmed shape deformation due to an environmental stimulus such as temperature (5), pH (6), or light (7). Conventionally studied stimuli-responsive composite hydrogels are typically composed of a synthetic stimuli-responsive hydrogel [i.e., poly(N-isopropylacrylamide) (PNIPAm) (4, 5) or poly(carboxylic acid) (8) and a synthetic additive [i.e., graphene oxide (4), carbon nanotubes (9), or metallic nanoparticles (10). Generally, the chosen additives imbue additional responsive behavior to these synthetic, stimuli-responsive composite hydrogel systems (11). For example, when a photothermal additive (e.g., graphene oxide) is added to temperature-responsive PNIPAm, the resulting stimuli-responsive composite hydrogel responds to both near-infrared radiation and temperature (4). While synthetic additives have proven successful in endowing existing stimuli-responsive hydrogels with additional responsive behaviors, these purely synthetic systems are still hindered by their inability to evolve with time. Because synthetic systems do not change with time, they have a finite number of equilibrium conditions in which they can be studied. The study of materials in nonequilibrium conditions, such as those arising from cell growth, provides insight into their potential behaviors and applications across various scenarios, including biomolecule transport through porous membranes and cell motility on the surface of materials (12).

Recent studies have utilized microorganisms as additives for hydrogel systems to engineer dynamic composite materials that can change with time. These microorganism-laden hydrogels are part of the emerging field of engineered living materials (ELMs), which explores the intersection between living cells and abiotic materials to create dynamic composites that harness the biological power of cells and exploit their functionality (13). Previously studied ELMs include cell-based contamination sensors (14, 15), antibiotic resistant surfaces (16), cell-laden bioinks (17, 18), sweat-sensing wearables (19), self-repairing concrete (20), and hydrogels for bioremediation of polluted water (21). Overall, ELMs hold promise in a broad range of applications ranging from soft robotics to biosensors.

Soft robotic-inspired, stimuli-responsive ELMs are a subclass of ELMs capable of shape-morphing in response to specific environmental or biochemical cues (22). Previous studies have shown that stimuli-responsive ELMs can respond to various inputs [e.g., biomolecules (18), light (23), and magnetic fields (24); however, their shape-morphing response is often less complex when compared to synthetic hydrogels (18). This is because shape-morphing mechanisms from existing ELMs rely mostly on significant and irreversible changes to the volume and swelling ratios from rapid cellular proliferation (18, 23, 25), whereas synthetic stimuli-responsive hydrogels undergo shape-morphing based upon reversibly altering its microstructure in response to environmental stimuli. For example, Rivera-Tarazona and coworkers demonstrated an irreversible and programmable shape change of a flat disc to a hat-like structure driven by rapid cell growth of *Saccharomyces cerevisiae* embedded within polyacrylamide (23). In contrast, Zhao and Bae have demonstrated that temperature-responsive nanocomposite nanoclay poly(N-isopropylacrylamide) can undergo reversible and programmable shape-morphing from a flat three-paneled sheet into a folded box-structure (5). While microorganisms (19, 21) can impart responsive behavior onto otherwise non-stimuli-responsive materials, their shape-morphing response is relatively limited compared to synthetic-based stimuli-responsive composite hydrogels (26).

The limited shape-morphing response displayed by stimuli-responsive ELMs can be attributed to their composition. Previously studied stimuli-responsive ELMs have relied solely on microorganisms to provide the stimuli-responsive behavior (17, 19, 23). To address this gap in the functionality of both stimuli-responsive ELMs and synthetic stimuli-responsive composite hydrogels, microorganisms with innate stimuli-responsive characteristics can be added to synthetic hydrogels with desirable stimuli-responsive capabilities, resulting in ELMs capable of changing with time and responding more dramatically to a variety of stimuli.

Here, we present an approach to preparing a stimuli-responsive ELM composed of the temperature-responsive hydrogel poly(N-isopropylacrylamide) (PNIPAm), rheological modifier Laponite nanoclay (NC), and the photosynthetic cyanobacterium *Synechococcus elongatus* sp. PCC 7942 (*S. elongatus*). PNIPAm is a well-studied, biocompatible (27), temperature-responsive hydrogel known for its ability to reversibly swell and deswell when its aqueous environment passes through its lower critical solution temperature (LCST) ranging from 30 to 50 °C (28). NC is a biocompatible disc (≈25 nm diameter and ≈1 nm thick) (29) that has utility as a rheological modifier for precursor solutions (30). In combination, NC-PNIPAm is a nanocomposite hydrogel that retains the temperature-responsive properties of PNIPAm while modifying its rheological behavior to facilitate more controllable casting and 3D printing of structures (5). The cyanobacterium *S. elongatus* was selected for this ELM system due to its photosynthetic metabolism (31), ability to biodegrade chemical pollutants (32), genetic tractability, and viability both above and below the LCST of NC-PNIPAm (33). This ELM has the capacity to reversibly change shape due to temperature, irreversibly change its curvature and local Young’s modulus over time, and respond to additional stimuli based on natural or engineered biology of *S. elongatus*. In creating this ELM, we demonstrate a diffusion mechanism that enables the incorporation of cyanobacterial cells into NC-PNIPAm despite the toxicity of the precursor solution. This method exploits the LCST of NC-PNIPAm to form a stimuli-responsive ELM, NC-PNIPAm/Se, that can undergo reversible shape deformation using a temperature stimulus. Additionally, we show that irreversible shape changes in the ELM are catalyzed by a previously unannotated *S. elongatus* amidase, AmiX. The creation and observation of this ELM informs construction of ELMs prepared from toxic precursors and highlights the potential of cell–material interactions under nonequilibrium conditions for the identification of properties such as continuously mechanically evolving materials.

## Results

### Preparation of NC-PNIPAm/Se via Temperature-Dependent Diffusion.

Previously studied stimuli-responsive ELMs employ a one-pot mixing approach to prepare samples (17, 18, 21), where living cells are directly added into a precursor solution before crosslinking. However, this approach is not transferable for preparing NC-PNIPAm/Se. When cultures of *S. elongatus*, composed of cyanobacterial medium blue-green-11 (BG-11) inoculated with *S. elongatus* cells, are incorporated into the precursor solution of NC-PNIPAm, it results in unhealthy and dying cells within 1 h of mixing (*SI Appendix*, Fig. S1*A*). Upon curing NC-PNIPAm/Se prepared by one-pot-mixing, the sample showed yellowing consistent with chlorosis over 7 d of incubation (*SI Appendix*, Fig. S1 *B* and *C*). When autofluorescence was compared to healthy *S. elongatus* cells on BG-11 agar plates, the cells exposed to the precursor solution showed abnormal internal organization and were considered nonviable (*SI Appendix*, Fig. S1*D*). This outcome likely results from exposure to high concentrations of N-isopropylacrylamide (NIPAm) monomer, a known cytotoxin (34), within the precursor solution, therefore making the one-pot mixing preparation unfeasible.

**Fig. 1.**
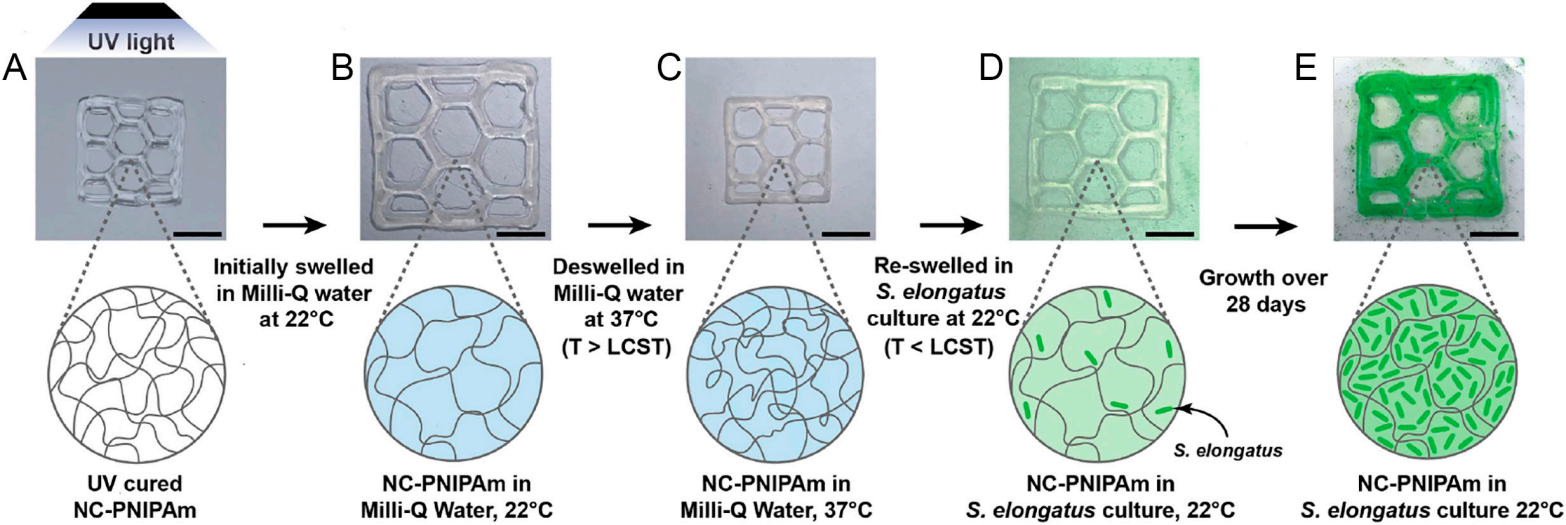
Diffusion of *S. elongatus* cells into NC-PNIPAm hydrogel. Optical images and corresponding illustration demonstrate the diffusion process used to introduce *S. elongatus* into NC-PNIPAm structures. Optical images show the 3D printed NC-PNIPAm structure (10 mm × 10 mm × 1 mm, 9% infill honeycomb). (*A*) NC-PNIPAm structure crosslinked using UV light at 60 mW/cm^2^ for 2 min, (*B*) NC-PNIPAm fully swelled in Milli-Q water at 22 °C below the LCST, (*C*) NC-PNIPAm fully deswelled in Milli-Q water at 37 °C above the LCST, (*D*) NC-PNIPAm reswelled at 22 °C in a culture of *S. elongatus* with cells diffused into the hydrogel, (*E*) NC-PNIPAm/Se ELM after 28 d of growth. (Scale bar, 5 mm.)

While the NC-PNIPAm precursor solution is toxic to cells, its polymerized counterpart, NC-PNIPAm, is biocompatible (27). Therefore, we opted to embed *S. elongatus* cells into NC-PNIPAm after crosslinking was completed by exploiting the temperature-controlled swelling and deswelling behavior causing expansion and contraction of NC-PNIPAm, respectively. This means of introducing live cells into the hydrogel matrix was inspired by previous studies showing that microorganisms can traverse and escape hydrogel matrices (18, 25). When heated from room temperature (22 °C) to 32 °C, NC-PNIPAm exhibits a reversible volume transformation where the hydrogel network transitions from a hydrophilic swelled state to a hydrophobic deswelled state (28). We exploited the transition of NC-PNIPAm from the deswelled state to the swelled state to incorporate *S. elongatus* cells into a fully crosslinked, 3D printed sample of NC-PNIPAm. These samples were fabricated using 3D printing to facilitate growth of *S. elongatus*. Datta and coworkers noted enhanced growth of *S. elongatus* cells embedded within sodium alginate when 3D printed into structures with high surface area to volume ratio (21). Immediately after crosslinking (Fig. 1*A*) the NC-PNIPAm was swelled in Milli-Q water at 22 °C (Fig. 1*B*) and then incubated in Milli-Q water at 37 °C to deswell and purge the water (Fig. 1*C*). To incorporate the *S. elongatus* cells into NC-PNIPAm, the deswelled NC-PNIPAm was transferred to a culture of *S. elongatus* cells at 22 °C to reswell, allowing the *S. elongatus* cells and their carrier solution, BG-11 growth medium, to diffuse into the hydrogel matrix (Fig. 1*D*). After the NC-PNIPAm was reswelled in the culture of *S. elongatus* cells, the stimuli-responsive ELM, NC-PNIPAm/Se, was transferred to BG-11 growth medium and allowed to grow for 28 d (Fig. 1*E*; *Materials and Methods*). We note that the slight size difference between the NC-PNIPAm fully swelled in Milli-Q water (Fig. 1*B*) and NC-PNIPAm/Se (Fig. 1*D*) can be attributed to the small difference in the linear swelling ratio, a measured property that is used to understand differences in water absorption of hydrogels (35). The linear swelling ratio was calculated as the ratio between the diameter of a fully swelled hydrogel disc divided by the diameter of the hydrogel in its as-prepared state. We compared the linear swelling ratios of NC-PNIPAm when swelled in three different solutions: Milli-Q water (1.623 ± 0.021), *S. elongatus* culture (1.488 ± 0.012), and BG-11 growth medium (1.460 ± 0.013), the liquid medium in which *S. elongatus* cells are dispersed. These differences between water and BG-11-containing samples are as expected, because this growth medium is an ionic solution optimized for cyanobacterial growth, and ionic solutions can decrease the linear swelling ratios of hydrogels (36).

### Viability of *S. elongatus* Cells Diffused Into NC-PNIPAm.

**Fig. 2.**
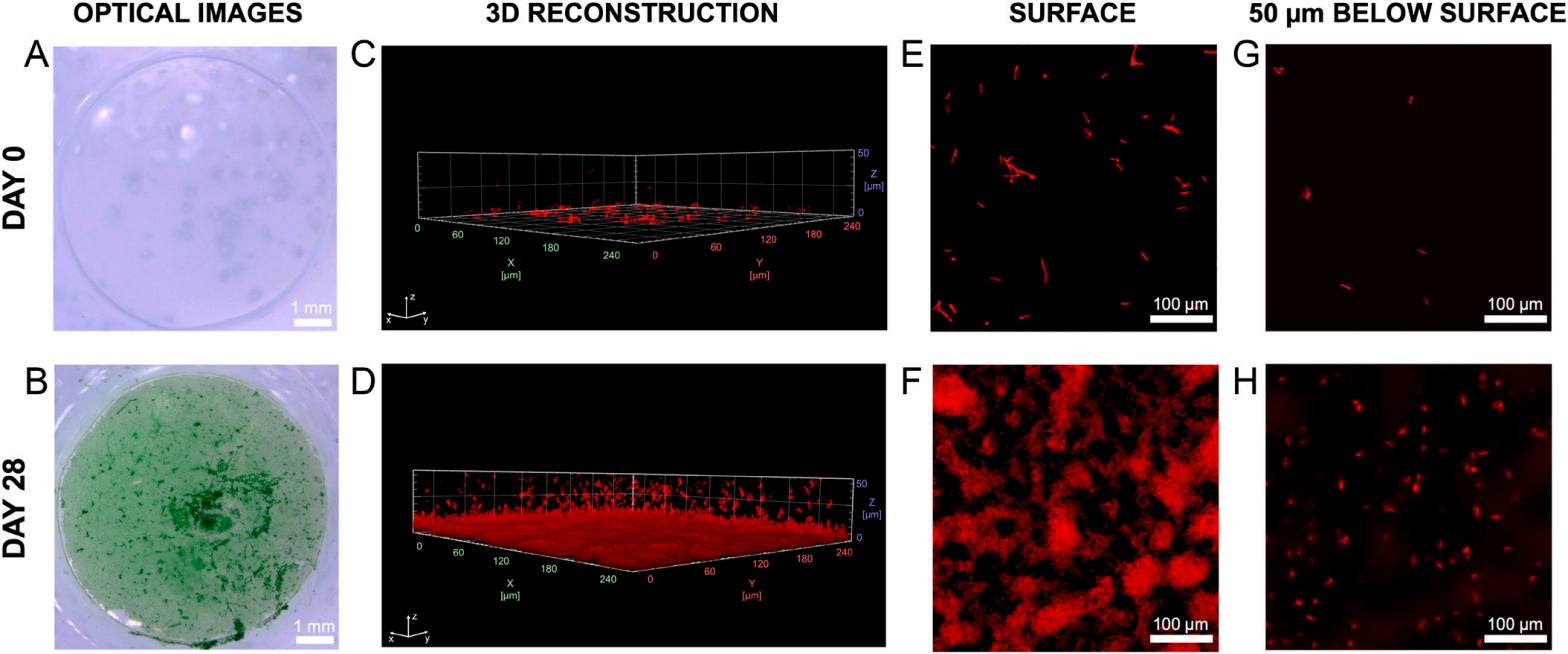
Casted discs of stimuli-responsive ELM NC-PNIPAm/Se imaged immediately after fabrication and after 28 d of growth. Optical images of NC-PNIPAm/Se acquired (*A*) immediately after fabrication, Day 0, and (*B*) after 28 d of growth, Day 28. Samples were rinsed in BG-11 medium before confocal imaging. 3D volume reconstruction of NC-PNIPAm/Se on (*C*) Day 0 and (*D*) Day 28 from confocal microscope images. (*E*–*H*) Confocal microscope images showing chlorophyll *a* autofluorescence of live cells at the surface of the disc of NC-PNIPAm/Se on (*E*) Day 0 and (*F*) Day 28, and 50 µm below the surface of the disc on (*G*) Day 0 and (*H*) Day 28.

Discs (8.95 mm diameter and 0.1 mm thickness) of NC-PNIPAm/Se were imaged using confocal fluorescence microscopy to evaluate the viability and growth of *S. elongatus* when diffused into NC-PNIPAm as described in Fig. 1 immediately after fabrication (Day 0; Fig. 2*A*), and after 28 d of incubation (Day 28; Fig. 2*B*). Chlorophyll *a* autofluorescence was used to detect living *S. elongatus* cells within hydrogels (37). Based on images from confocal fluorescence microscopy, viable *S. elongatus* cells were detected on Day 0 (Fig. 2 *C*, *E*, and *G*) and on Day 28 (Fig. 2 *D*, *F*, and *H*) within NC-PNIPAm/Se, with a dramatic increase in the number of viable cells over that period. We observed a gradient in cell density within NC-PNIPAm/Se on Day 0, with a larger population of *S. elongatus* cells toward the outer surfaces and a smaller population of *S. elongatus* cells toward the center (Fig. 2 *C*, *E*, and *G*). A similar gradient in cell density was observed on Day 28, although the overall quantity of cells throughout the NC-PNIPAm was higher (Fig. 2 *D*, *F*, and *H*). We hypothesize that *S. elongatus* cell reproduction is most robust near the surface of NC-PNIPAm due to increased light penetration and gas exchange (21), and cells may colonize near the surface more frequently because they are introduced into the matrix from the outside.

Additionally, we tested two alternative diffusion methodologies that lacked the swelling and/or deswelling steps and monitored cell viability by confocal fluorescence microscopy. For the first alternative diffusion method, NC-PNIPAm was placed into a culture of *S. elongatus* immediately after crosslinking (*SI Appendix*, Fig. S2*A*). This method showed adequate cell viability immediately after fabrication both at the surface and 25 µm below the surface (Day 0; *SI Appendix*, Fig. S2 *B* and *D*), respectively, but did not support a similar degree of cellular proliferation after 28 d of incubation as when the steps of swelling and deswelling in Milli-Q water were included (Day 28; *SI Appendix*, Fig. S2 *C* and *E*). We hypothesize that the cytotoxic, residual, unreacted NIPAm monomer within the hydrogel inhibited growth of *S. elongatus* (34). For the second alternative diffusion method, NC-PNIPAm was preswelled in Milli-Q water, then transferred directly into a culture of *S. elongatus* at 22 °C (*SI Appendix*, Fig. S2*F*). Confocal fluorescence microscopy acquired immediately after fabrication (Day 0; *SI Appendix*, Fig. S2 *G* and *I*) and 28 d later (Day 28; *SI Appendix*, Fig. S2 *H* and *J*) showed that *S. elongatus* cells were unable to form colonies within the hydrogel matrix when NC-PNIPAm was preswelled with Milli-Q water. We concluded that the deswelling step is necessary for the creation of the ELM because it purges unreacted NIPAm monomer and water from the hydrogel matrix, preparing it to absorb *S. elongatus* cells in the reswelling step (Fig. 1). Thus, the deswelling of NC-PNIPAm and subsequent reswelling of NC-PNIPAm in *S. elongatus* culture are both crucial steps in our diffusion mechanism (Fig. 1).

Finally, we tested a third alternative diffusion method generalizable for a wide variety of hydrogels, that utilized the information gained from the first two alternative diffusion methods. For this, the UV crosslinked NC-PNIPAm was first purged of excess NIPAm monomer by swelling in Milli-Q water at 22 °C, deswelling in Milli-Q water 37 °C, and reswelling in Milli-Q water 22 °C (*SI Appendix*, Fig. S2*K*). It is expected that to achieve growth in most hydrogels reacted from toxic monomers, hydrogels would have to be washed with distilled water to remove unreacted monomers (38). After initially purging the NC-PNIPAm of excess NIPAm monomer, the NC-PNIPAm was completely dehydrated in a convection oven at 65 °C overnight and then rehydrated in *S. elongatus* culture (*SI Appendix*, Fig. S2*K*). This diffusion method showed reasonable cell viability and some cell penetration at the surface and 25 µm below the surface after fabrication (Day 0; *SI Appendix*, Fig. S2 *L* and *N*), respectively. After incubation for 28 d, it showed decent cell proliferation on the surface, with less growth 25 µm within the hydrogel (Day 28; *SI Appendix*, Fig. S2 *M* and *O*), respectively. Despite this generalized diffusion method showing modest cell growth and colonization, overall less growth was observed compared with our proposed diffusion mechanism (Fig. 2). We hypothesize that this reduced colonization and proliferation by *S. elongatus* cells within the rehydrated hydrogel is due to the NC-PNIPAm surface becoming less porous during the oven-drying process (39). To minimize the adverse effects oven drying has on hydrogels for a generalizable diffusion strategy, hydrogels could instead be dried partially before rehydrating in a cell culture solution, which would be more analogous to the temperature-dependent deswelling of NC-PNIPAm, where some, but not all, water is expelled from the hydrogel matrix.

### Experimental Diffusion Coefficient of *S. elongatus* Cells Entering NC-PNIPAm.

To ensure the feasibility of our proposed diffusion mechanism (Fig. 1), we first compared the average length of *S. elongatus* cells with the average pore size of NC-PNIPAm swelled in BG-11 media by Feret’s diameter, the measured distance between two restricted parallel planes in its perpendicular direction (40). This was executed by freeze-drying NC-PNIPAm swelled in BG-11 media and imaging the cross section using scanning electron microscopy (SEM). These images were measured using thresholding analysis in FIJI showing the NC-PNIPAm swelled in BG-11 had an average pore size of 17.8 ± 3.8 µm as measured by Feret’s diameter (*SI Appendix*, Fig. S3). The pore size of NC-PNIPAm swelled in BG-11 was compared with the average length of *S. elongatus* of approximately 2 µm (21). Overall, the average size of the NC-PNIPAm pores swelled in BG-11 exceeds the length of *S. elongatus* cells.

Furthermore, we investigated the diffusion coefficient of *S. elongatus* cells as they diffuse into NC-PNIPAm via the proposed mechanism. Generally, diffusion coefficients are important to characterize and understand the motion of particles across different interfaces. Previous studies have obtained experimental diffusion coefficients for solid particles obeying Brownian motion as they diffuse through both temperature-responsive and nonresponsive hydrogel matrices utilizing a statistical analysis technique, mean squared displacement (MSD) (41, 42). This analysis technique is rooted in the correlation described by the following equation, where *x* represents the displacement, *d* represents the number of dimensions through which diffusion takes place, *D_e_* represents the experimental diffusion coefficient, and τ represents the time it takes for the particles to be displaced (43).

$$\mathrm{MSD}\left(\tau \right)=\;<x^{2}>\;=2dD_{e}\tau ,$$

MSD analysis results in an experimental diffusion coefficient by analyzing the vector displacement of a single solid particle that abides by Brownian motion with respect to time. We note that our strain of *S. elongatus* does not form biofilms and is nonmotile, resulting in a pseudohomogeneous mixture wherein the *S. elongatus* cells obey Brownian motion (44). To obtain an experimental diffusion coefficient of *S. elongatus* cells, it was necessary to track the vector displacement of cells over time as they diffuse into NC-PNIPAm and then linearly fit the vector displacement of cells over time to the MSD equation. Once the datasets are fit to the MSD equation, the resulting slope is the experimental diffusion coefficient for *S. elongatus* cells diffusing into NC-PNIPAm transitioning from its contracted deswelled state to its expanded swelled state.

**Fig. 3.**
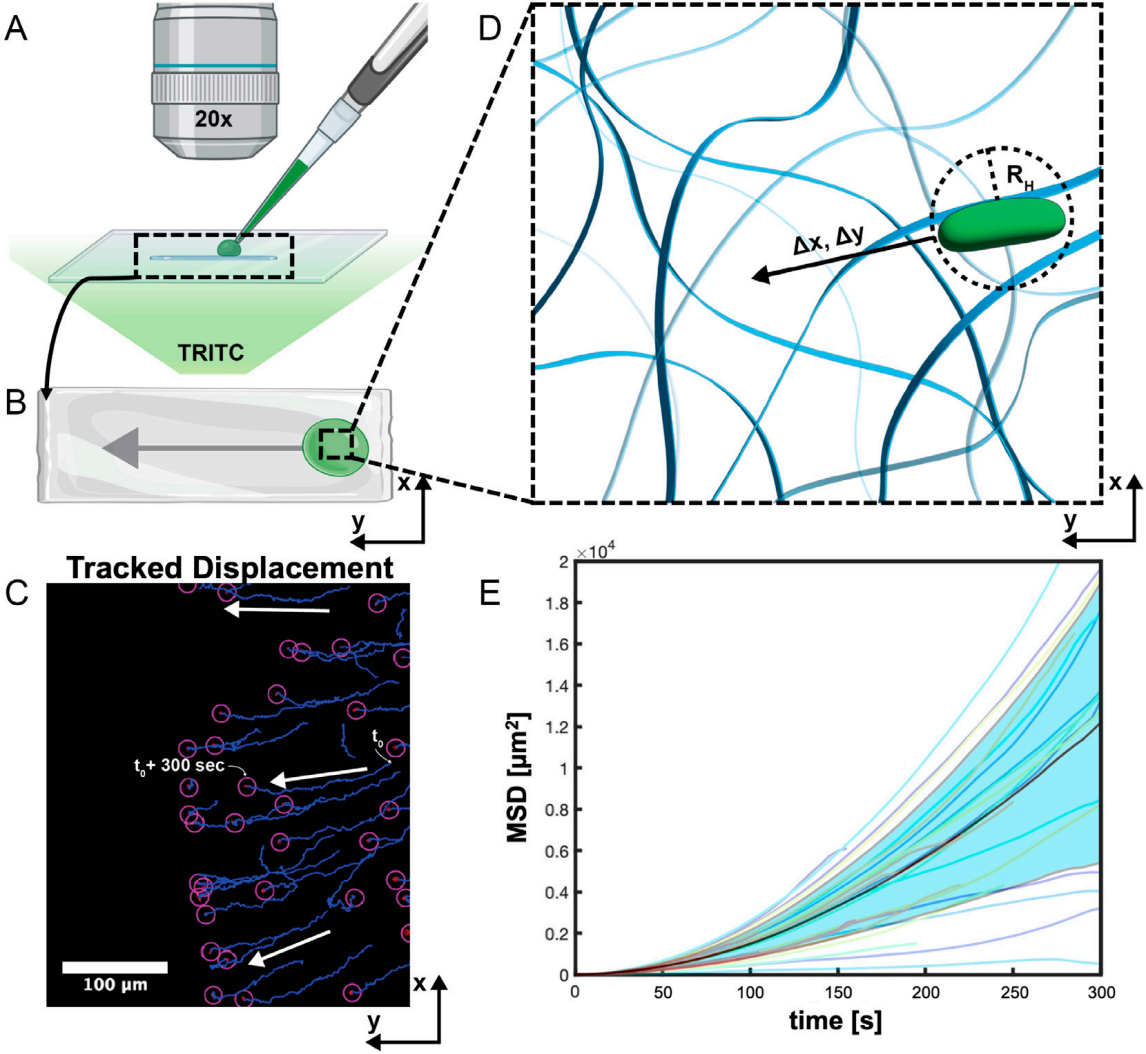
Experimental diffusion coefficient of *S. elongatus* cells entering NC-PNIPAm using MSD. (*A* and *B*) Illustrations depicting diffusion experiment. (*A*) Experimental set up where 5 µL of diluted culture of *S. elongatus* is pipetted onto the surface of deswelled NC-PNIPAm (20 mm × 5 mm × 0.025 mm) affixed onto a microscope slide. (*B*) Overhead view of deswelled NC-PNIPAm and droplet of *S. elongatus*; the arrow indicates direction cells move as diffusion takes place. (*C*) Schematic depicting microscopic movement of *S. elongatus* cells diffusing through two-dimensional hydrogel matrix as the NC-PNIPAm transitions from the deswelled to reswelled state. (*D*) Fluorescent image depicts *S. elongatus* cells detected by chlorophyll *a* autofluorescence with TRITC filter immediately after pipetting culture of *S. elongatus* onto the surface of NC-PNIPAm (denoted as t_0_) and 300 s after addition of culture of *S. elongatus* (denoted as t_0_ + 300 s). Purple circles illustrate the located cells and blue lines indicate the tracked vector displacement of cells, identified by multiple particle tracking FIJI plugin, respectively. White arrows indicate the general direction that *S. elongatus* cells move during the diffusion process. (*E*) MSD plotted as a function of time traveled and fitted to calculate experimental diffusion coefficient. MSD of individual *S. elongatus* cells are represented by colored lines, and the average MSD and SD of *S. elongatus* cells represented by the black line and light blue shaded region, respectively.

We measured the vector displacement of *S. elongatus* cells from a series of fluorescent images taken while *S. elongatus* cells were diffusing into NC-PNIPAm to determine the experimental diffusion coefficient. This was accomplished by placing an ultrathin deswelled sheet of NC-PNIPAm (20 mm × 5 mm × 0.025 mm) affixed to a microscope slide on a fluorescence microscope. To the deswelled sheet of NC-PNIPAm, a 5 µL droplet of a diluted culture of *S. elongatus* was pipetted onto the surface. As the NC-PNIPAm cooled down it transitioned from its contracted deswelled state to its expanded swelled state, absorbing the *S. elongatus* culture (Fig. 3 *A*–*C*). We note that due to the thinness of the hydrogel samples, we assumed the *S. elongatus* cell diffusion occurred only in two (*x-* and *y-*) dimensions as illustrated with the coordinate system in Fig. 3. *S. elongatus* cells diffusing through the NC-PNIPAm were captured beneath the NC-PNIPAm surface using a series of fluorescent images, the first taken just after pipetting *S. elongatus* onto the surface of NC-PNIPAm (t_0_) and continuing in 5 s intervals until reaching 300 seconds (t_0_ + 300 s) (Fig. 3*D* and Movie S1). The images were analyzed to quantify the vector displacement of each cell using an open-sourced multiple particle tracking FIJI plugin, TrackMate, developed by Ershov et al. (45). Utilizing the previous research of Tarantino et al. (46), the tracked cells and their vector displacement were analyzed in an MSD analyzer in MATLAB, and the tracked cells and their vector displacement were linearly fit to the MSD equation (Fig. 3*E*). Its resulting slope, the experimental diffusion coefficient (*D_e_*) for *S. elongatus* cells entering NC-PNIPAm as it transitions from its deswelled to swelled state was 9.5 × 10^−12^ ± 8.1 × 10^−12^ m^2^/s. Notably, the experimental diffusion coefficient of *S. elongatus* cells is one order of magnitude smaller than the poroelastic diffusion of water into PNIPAm hydrogels (4), 1 × 10^−11^ m^2^/s, likely due to the culture of *S. elongatus* being composed of a low concentration of *S. elongatus* cells dispersed in BG-11 medium, an aqueous, ionic solution. We compared this experimental diffusion coefficient (*D_e_*) with a theoretical diffusion coefficient (*D_t_*) calculated using the Stokes–Einstein equation derived for two-dimensions, modified for rod-shaped particles due to the rod-like shape of *S. elongatus* cells, as previously demonstrated by Wang and coworkers (41, 47, 48) (*Materials and Methods*). The resulting theoretical diffusion coefficient, *D_t_*, of an *S. elongatus* cell entering the NC-PNIPAm hydrogel matrix as it transitions from a deswelled to a swelled state was calculated as 1.1 × 10^−13^ m^2^/s. We hypothesize that the one order of magnitude difference between the experimental value and theoretical value is due to our model not accounting for surface crossing effects, which can cause artificially elevated experimental diffusion coefficients (49).

### Changes in Bending Curvature and Modulus of NC-PNIPAm/Se Under Nonequilibrium Conditions.

**Fig. 4.**
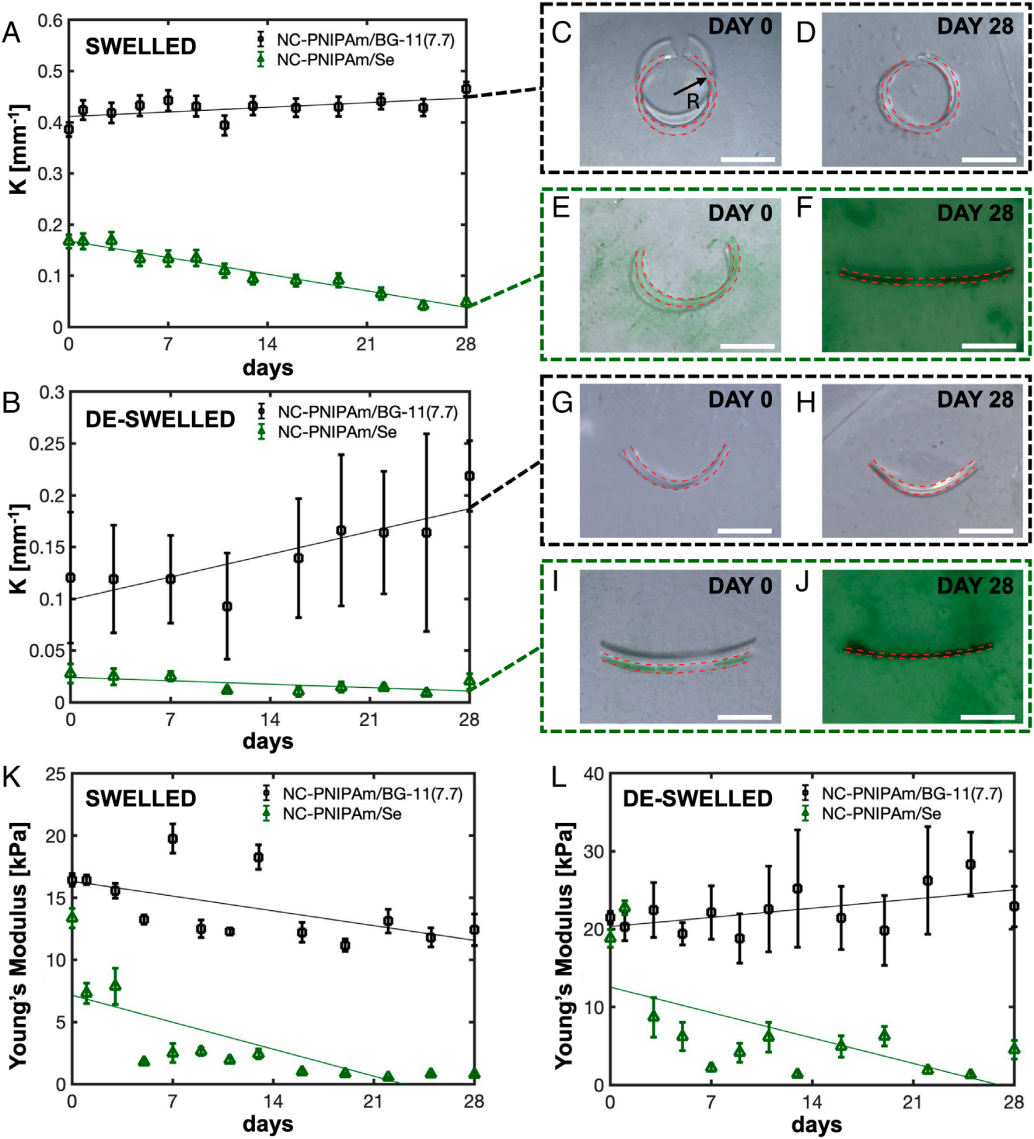
Changes in bending curvature and local Young’s modulus over time of swelled and deswelled NC-PNIPAm, with and without incorporated *S. elongatus* cells. Bending curvature of NC-PNIPAm/Se (10 mm × 2.5 mm × 0.025 mm, shown in green) compared to NC-PNIPAm diffused with a BG-11 control without cells (10 mm × 2.5 mm × 0.025 mm, NC-PNIPAm/BG-11(7.7), shown in black) in (*A*) swelled and (*B*) deswelled states. Green (r^2^ = 0.96, r^2^ = 0.43) and black (r^2^ = 0.35, r^2^ = 0.68) lines indicate linear regression curves for bending curvature data in swelled and deswelled states of NC-PNIPAm/Se and NC-PNIPAm/BG-11, respectively. (*C*) Bending curvature *K* was calculated using the equation *K = 1/R*, where *R* represents the radius of curvature measured from the inner radius. (*C*–*J*) Photographs show bending curvature of swelled NC-PNIPAm/BG-11(7.7) from (*C*) Day 0 to (*D*) Day 28, swelled NC-PNIPAm/Se from (*E*) Day 0 to (*F*) Day 28; deswelled NC-PNIPAm/BG-11(7.7) from (*G*) Day 0 to (*H*) Day 28, and deswelled NC-PNIPAm/Se from (*I*) Day 0 to (*J*) Day 28. The observed changes in bending curvature values are summarized in *SI Appendix*, Table S1, where each data point averages five measurements. Local Young’s moduli of NC-PNIPAm/Se and NC-PNIPAm/BG-11(7.7) from Day 0 to Day 28 in their (*K*) swelled and (*L*) deswelled states. Green (r^2^ = 0.56, r^2^ = 0.42) and black (r^2^ = 0.33, r^2^ = 0.31) lines indicate linear regression curves for local Young’s modulus data in swelled and deswelled states of NC-PNIPAm/Se and NC-PNIPAm/BG-11, respectively. The observed changes in the local Young’s modulus values are summarized in *SI Appendix*, Table S4, where each data point averages the local Young’s modulus of a minimum of 10 measurements. (Scale bars, 5 mm.)

When using the diffusion mechanism illustrated in Fig. 1 to fabricate thin sheets (10 mm × 2.5 mm × 0.025 mm) of NC-PNIPAm/Se, we observed bending curvature changes in both the swelled and deswelled states over a 28-d observation period (Fig. 4 *A* and *B* and *SI Appendix*, Table S1). These samples were measured periodically throughout a 28-d observation period in their swelled and deswelled states because the ELM was expected to change over time under nonequilibrium conditions in response to cell growth, and because NC-PNIPAm can undergo reversible shape deformation in response to temperature (28). As a cell-free control, BG-11 growth medium was buffered to pH = 7.7 and diffused into NC-PNIPAm, denoted as NC-PNIPAm/BG-11(7.7). The abbreviations for NC-PNIPAm diffused with different media are defined and summarized in *SI Appendix*, Table S2. We report the bending curvature as *K = 1/R*, where *R* represents the radius of curvature, which was measured along the inner radius of the hydrogel sheet (Fig. 4*C*). Over 28 d, the bending curvature of NC-PNIPAm/BG-11(7.7) increased by 20% (Fig. 4 *C* and *D* and *SI Appendix*, Table S1). In contrast, the bending curvature of NC-PNIPAm/Se decreased by 71% over 28 d (Fig. 4 *E* and *F* and *SI Appendix*, Table S1). Additionally, hydrogels without a bilayer do not typically exhibit a high degree of curvature when swelled (50). However, when samples are very thin and there is sufficient shear stress during the fabrication process, hydrogels can exhibit a high degree of bending curvature in their swelled state without the presence of a bilayer and anisotropic swelling (50). Additionally, previous studies have demonstrated that exposure to UV light on a singular side can cause a gradient of photopolymerization with a higher degree of crosslinking closer to the UV light source, therefore causing bending in otherwise chemically homogeneous polymers (51). Samples of NC-PNIPAm were maintained in the swelled state until their prescribed deswelling date, upon which the samples were deswelled for 2 h at 37 °C, and their bending curvatures were measured. The bending curvature of NC-PNIPAm can change slightly when repeatedly cycled between swelled and deswelled states (52). To eliminate the contribution of cycling to observed changes in shape, each sample was deswelled and measured only once. The LCST range of NC-PNIPAm/Se was tested and compared to NC-PNIPAm/BG-11(7.7) immediately after sample preparation (Day 0), and after 28 d of observation (Day 28) to ensure that it remained at the literature value of approximately 31 to 33 °C (28) for proper deswelling (*SI Appendix*, Fig. S4). In its deswelled state, the bending curvature of NC-PNIPAm/BG-11(7.7) increased by 83% over 28 d (Fig. 4 *G* and *H* and *SI Appendix*, Table S1). In comparison, the bending curvature of deswelled NC-PNIPAm/Se decreased by 68% over 28 d (Fig. 4 *I* and *J* and *SI Appendix*, Table S1). The higher SD in deswelled curvature measurements can be attributed to temperature instability due to heat loss experienced during the measuring process (*SI Appendix*, Table S1). This experiment was repeated in both swelled and deswelled states with 100 µm thick samples of NC-PNIPAm, which exhibited the same trend, albeit with less dramatic bending curvature changes (*SI Appendix*, Fig. S5), likely due to the higher bending energy required to observe changes in the bending curvature of thicker samples (53).

To determine the cause of the observed decrease in bending curvature of NC-PNIPAm/Se compared to NC-PNIPAm/BG-11(7.7), we investigated their linear swelling ratio, linear deswelling ratio, and local Young’s modulus near the gel surface, as these characteristics typically explain differing bending curvature in hydrogels (54). The linear swelling ratio was calculated using $\lambda_{s}=D_{s}/D_{0}$ and the linear deswelling ratio was calculated using $\lambda_{d}=D_{d}/D_{0}$, where *D_s_* represents the diameter of a fully swelled hydrogel, *D_d_* represents the diameter of a fully deswelled hydrogel, and *D_0_* represents the diameter of the hydrogel as prepared. NC-PNIPAm/Se and NC-PNIPAm/BG-11(7.7) both exhibited minimal changes in the linear swelling ratio and deswelling ratio (*SI Appendix*, Fig. S6 and Table S3). The linear swelling ratio of NC-PNIPAm/Se and NC-PNIPAm/BG-11(7.7) both slightly increased by 9% and 7%, respectively, over the 28-d observation period. Similarly, the linear deswelling ratios for NC-PNIPAm/Se and NC-PNIPAm/BG-11(7.7) both slightly increased over the 28-d observation period by 8% and 11%, respectively.

While the linear swelling ratio slightly increased with time, the local Young’s modulus dramatically decreased over 28 d for both swelled and deswelled NC-PNIPAm/Se, dropping by 94% and 76% in its swelled and deswelled states, respectively (*SI Appendix*, Fig. S6). In contrast, the local Young’s modulus of NC-PNIPAm/BG-11(7.7) decreased less in its swelled state over 28 d, by 24%, and increased in its deswelled state over the same period, by 7% (Fig. 4 *K* and *L* and *SI Appendix*, Table S4). We note that the margin of error for the local Young’s modulus measurements is large due to measuring highly localized regions with a nanoindenter. From observation of the growth rate and distribution of *S. elongatus* cells embedded in NC-PNIPAm (Fig. 2) and the decrease in the local Young’s modulus of NC-PNIPAm/Se, we hypothesize that by Day 28, the NC-PNIPAm/Se ELM contains a pseudo-bilayer with a high cell count and low corresponding local Young’s modulus toward the surface, and a low cell count and high corresponding local Young’s modulus toward the center. Battista and coworkers have shown through computational analysis that bilayer hydrogel structures with mismatched Young’s moduli can experience reduced curvature or flatten over time (55). We observed that *S. elongatus* cell density increases disproportionately toward the surface (Fig. 2); therefore, it likely results in an increasingly mismatched local Young’s modulus, causing the bending curvature of NC-PNIPAm/Se to decrease over time under nonequilibrium conditions.

### Partial Enzymatic Degradation of NC-PNIPAm.

*S. elongatus* cells are capable of producing and secreting numerous molecules and proteins into their surrounding environment (56). We hypothesized that an enzyme(s) produced by *S. elongatus* that alters the hydrogel matrix might account for the observed change in curvature of the ELM. Enzymes secreted from cells are known to cleave bonds within synthetic hydrogels, such as polyacrylamide (57). To test secreted products of *S. elongatus* for the ability to degrade NC-PNIPAm, we prepared conditioned medium by removing *S. elongatus* cells from a three-week-old culture by centrifugation and filtration, leaving the growth medium and the proteins and molecules secreted by cells, as well as those present due to cell lysis. Because growth of *S. elongatus* gradually increases the pH of the growth medium, we also explored the effect of changing pH on NC-PNIPAm by buffering 15 mL aliquots of the same conditioned medium to a low pH condition (pH = 7.7) and a high pH condition (pH = 10.0) using 1 M Tris-HCl (pH = 7.5) and 0.8 M glycine (pH = 10.0), respectively. As a negative control for most enzyme activity, another aliquot of conditioned medium was protease-treated and autoclaved (*SI Appendix*, *Preparation of Conditioned Media*). The low pH conditioned medium, high pH conditioned medium, and autoclaved conditioned medium were each diffused into NC-PNIPAm, denoted as NC-PNIPAm/CM(7.7), NC-PNIPAm/CM(10), and NC-PNIPAm/CM(Aclv), respectively (*SI Appendix*, Table S2), to test the bending curvature and local Young’s modulus over time and determine whether or not they emulated the changes brought about by the growth of *S. elongatus* in NC-PNIPAm.

This preliminary investigation demonstrated that both NC-PNIPAm/CM(7.7) and NC-PNIPAm/CM(10) exhibited a decreasing trend in the local Young’s modulus similar to NC-PNIPAm/Se in both the swelled and deswelled states, whereas the NC-PNIPAm/CM(Aclv) emulated the trend of the cell-free control, NC-PNIPAm/BG-11(7.7) (*SI Appendix*, Fig. S7 *A* and *B* and Table S4). The local Young’s modulus of NC-PNIPAm/CM(7.7) decreased over the 28-d observation period by 77% and 89% in its swelled and deswelled states, respectively. Similarly, the local Young’s modulus of NC-PNIPAm/CM(10) in its swelled and deswelled states decreased by 91% and 54%, respectively. In comparison, the local Young’s modulus of NC-PNIPAm/CM(Aclv) was relatively stable, increasing by 9% in its swelled state and decreasing by 17% in its deswelled state.

The bending curvatures of NC-PNIPAm/CM(7.7) and NC-PNIPAm/CM(10) were tested in the swelled state over 28 d of observation and decreased by 80% and 83%, respectively (*SI Appendix*, Fig. S7*C* and Table S1). When deswelled, the bending curvature of NC-PNIPAm/CM(7.7) decreased by 51% whereas NC-PNIPAm/CM(10) increased by 18% (*SI Appendix*, Fig. S7*D* and Table S1). We attribute this increase in deswelled bending curvature of NC-PNIPAm/CM(10) at day 28 to variation in sample preparation, which is a more prominent factor when the magnitude of the curvature change is small (*SI Appendix*, Fig. S7 *C* and *D* and Table S1). The deswelled bending curvature values of NC-PNIPAm/CM(10) measured prior to Day 28 were consistently lower than the initial value of 0.044 ± 0.014 mm^−1^ (*SI Appendix*, Fig. S7*D*). The curvature of NC-PNIPAm/CM(Aclv) decreased slightly in both the swelled and deswelled states, by 2% and 19%, respectively (*SI Appendix*, Fig. S7 *C* and *D*). Although NC-PNIPAm exposed to conditioned media that contains potentially active enzymes experienced a decrease in bending curvature and local Young’s modulus at both pH = 7.7 and pH = 10.0, the magnitude of the change was slightly less than that observed with NC-PNIPAm/Se. We speculate that the change is greater in the presence of living cells due to their continuous secretion of proteins throughout the 28-d observation period, resulting in higher levels of enzymatic activity. Additionally the linear swelling ratios of NC-PNIPAm/CM(7.7), NC-PNIPAm/CM(10), and NC-PNIPAm/CM(Aclv) were relatively stable over time, increasing by 7%, 4%, and 1%, respectively (*SI Appendix*, Fig. S6*A* and Table S3). The linear deswelling ratios of NC-PNIPAm/CM(7.7), NC-PNIPAm/CM(10), and NC-PNIPAm/CM(Aclv) also remained relatively stable, increasing by 12%, 10%, and 12%, respectively (*SI Appendix*, Fig. S6*B* and Table S3).

To determine whether pH impacts the curvature and modulus of NC-PNIPAm independent of cells or cellular products, samples of BG-11 were buffered to pH = 7.7 and pH = 10.0 and diffused into NC-PNIPAm, denoted as NC-PNIPAm/BG-11(7.7) and NC-PNIPAm/BG-11(10), respectively. NC-PNIPAm/BG-11(10) exhibited a slight decrease in curvature, 12% and 10% in swelled and deswelled states, respectively (*SI Appendix*, Fig. S7 *C* and *D*). In contrast, the curvature of NC-PNIPAm/BG-11(7.7) increased over 28 d by 20% and 83% in swelled and deswelled states, respectively (Fig. 4 *A* and *B*). The local Young’s modulus of NC-PNIPAm/BG-11(10) remained relatively stable in the swelled state, increasing by 9%, and by a larger amount, 20%, in the deswelled state (*SI Appendix*, Fig. S7 *A* and *B*). The local Young’s modulus of NC-PNIPAm/BG-11(7.7) decreased by 24% in the swelled state and increased by 7% in the deswelled state (Fig. 4 *K* and *L*). The linear swelling and deswelling ratios of NC-PNIPAm/BG-11(10) remained relatively unchanged, decreasing by 9% and increasing by 2%, respectively. The linear swelling and deswelling ratios of NC-PNIPAm/BG-11(7.7) exhibited similarly slight increases, 7% and 11%, respectively (*SI Appendix*, Fig. S6). Elevated pH had a greater impact on curvature and the local Young’s modulus than on the linear swelling and deswelling ratios. However, the similarity of NC-PNIPAm/CM(7.7) and NC-PNIPAm/CM(10) with respect to curvature and local Young’s modulus over 28 d suggests that the observed enzymatic activity has a larger impact on these material characteristics than increasing the pH from 7.7 to 10.0. (Fig. 4 *A, B*, *K*, and *L* and *SI Appendix*, Fig. S7). Considering the more dramatic changes observed in bending curvature and local Young’s modulus between samples with and without potential enzymatic activity, we hypothesized that the observed changes in NC-PNIPAm/Se bending curvature and local Young’s modulus within 28 d are primarily due to an enzyme secreted by *S. elongatus*.

**Fig. 5.**
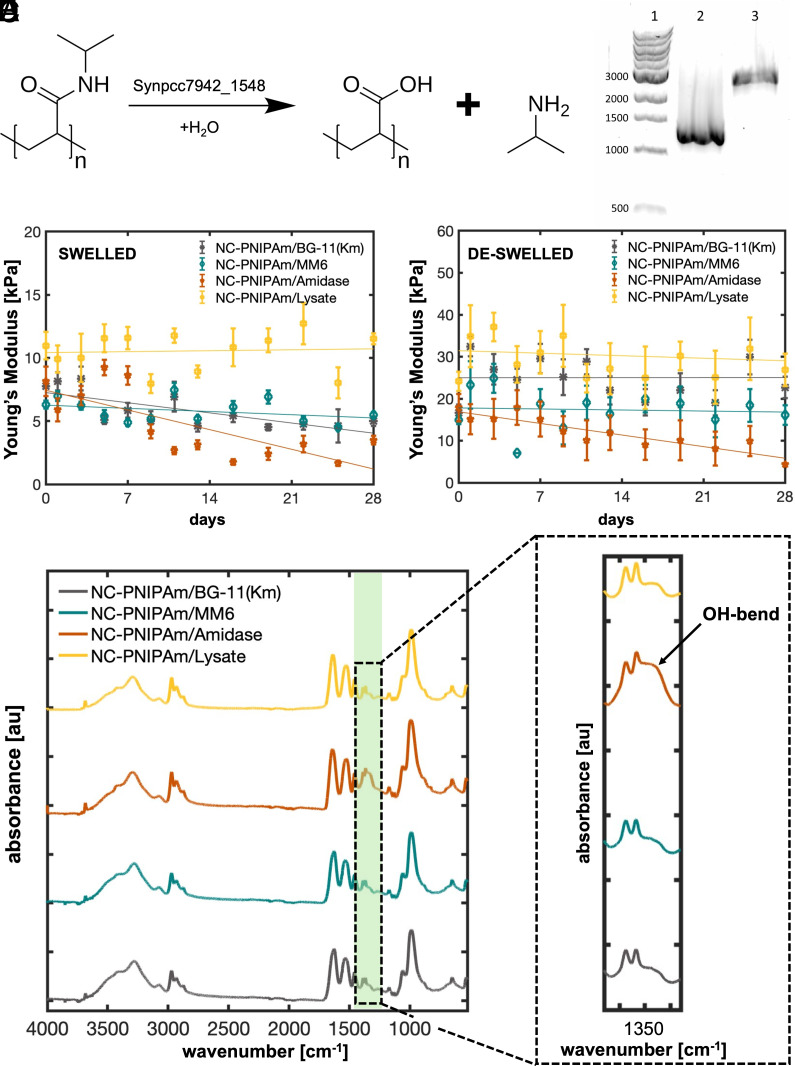
The effect of a putative *S. elongatus* amidase on NC-PNIPAm. (*A*) Proposed mechanism for the hydrolysis of amide bonds in NC-PNIPAm by Synpcc7942_1548, a putative amidase secreted by *S. elongatus* (60). (*B*) Products of PCR of the Synpcc7942_1548 open reading frame (*SI Appendix*, *Supporting Materials and Methods*) showing the size difference between the wild-type (lane 2; ~1,320 bp) and insertional mutant MM6 (lane 3; ~3,321 bp). Lane 1 is a labeled size marker showing DNA length in base pairs. No wild-type copies of the gene remain in strain MM6. (*C*) NC-PNIPAm local Young’s modulus in the swelled and (*D*) deswelled states after diffusion with BG-11 (pH = 7.7) containing one of the following: kanamycin (5 µg/mL; gray), the insertional mutant (MM6; teal), the purified, recombinant product of Synpcc7942_1548 expressed in *Escherichia coli* (45.5 µg/mL; orange), a control lysate from *E. coli* with no heterologous expression (45.5 µg/mL total protein; yellow). Gray (r^2^ = 0.54, r^2^ = 0.00), teal (r^2^ = 0.13, r^2^ = 0.00), orange (r^2^ = 0.57, r^2^ = 0.75), and yellow (r^2^ = 0.00, r^2^ = 0.03) lines indicate linear regression curves for local Young’s modulus data in the swelled and deswelled states (*SI Appendix*, Table S4). (*E* and *F*) Samples NC-PNIPAm/BG-11(Km) (gray), NC-PNIPAm/MM6 (teal), NC-PNIPAm/Amidase (orange), and NC-PNIPAm/Lysate (yellow) were incubated for 28 d, and their chemical composition was measured using ATR-FTIR. NC-PNIPAm/Amidase exhibits the OH-bend between wavenumbers 1,440 and 1,395 cm^−1^ characteristic of a carboxylic acid (black arrow).

The following six samples were analyzed by Attenuated Total Reflectance-Fourier Transform Infrared Radiation (ATR-FTIR) to test for products of enzyme-mediated hydrogel degradation: NC-PNIPAm/BG-11(7.7), NC-PNIPAm/BG-11(10), NC-PNIPAm/CM(7.7), NC-PNIPAm/CM(10), NC-PNIPAm/CM(Aclv), and NC-PNIPAm/Se. ATR-FTIR results showed that the three samples potentially containing active *S. elongatus* enzymes [NC-PNIPAm/Se, NC-PNIPAm/CM(7.7), and NC-PNIPAm/CM(10)] all exhibited a characteristic OH-bend between 1,440 and 1,395 (*SI Appendix*, Fig. S8 *A* and *B*), further implicating one or more enzymes in the observed decrease in bending curvature and local Young’s modulus. This OH-bend is indicative of a carboxylic acid, a functional group that is absent in samples with no cell products or with proteolyzed/autoclaved cell products (NC-PNIPAm/BG-11(7.7), NC-PNIPAm/BG-11(10), and NC-PNIPAm/CM(Aclv)). Amidases are a type of enzyme known to degrade amide bonds through amide hydrolysis, creating a carboxylic acid (58). Yegorov et al. identified the Synpcc7942_1548 gene product, annotated as a probable amidase, in the conditioned media of *S. elongatus* (59), suggesting that it is transported outside the cell. We hypothesized that this putative amidase cleaves amide bonds in NC-PNIPAm to form carboxylic acid and isopropylamine, thereby partially degrading NC-PNIPAm (Fig. 5*A*).

To test whether Synpcc7942_1548 is responsible for the proposed partial degradation of NC-PNIPAm, it was heterologously expressed in *E. coli*, purified (*SI Appendix*, *Preparation of Purified Amidase*), added to BG-11 (pH = 7.7; 45.5 µg/mL), and diffused into NC-PNIPAm. As a control, an insertional mutant incapable of producing the putative amidase, strain MM6, was constructed (*Materials and Methods*), verified (Fig. 5*B*), and then diffused into NC-PNIPAm. Strain MM6 was cultured in BG-11 medium with kanamycin (5 µg/mL) to maintain the gene insertion, which includes a kanamycin-resistance cassette. To test whether this concentration of kanamycin could impact the Young’s modulus of NC-PNIPAm, BG-11 with kanamycin (5 µg/mL) was diffused into NC-PNIPAm, denoted as NC-PNIPAm/BG-11(Km). These samples are denoted as NC-PNIPAm/Amidase, NC-PNIPAm/MM6, and NC-PNIPAm/BG-11(Km) respectively (*SI Appendix*, Table S2). The bending curvature, mechanical integrity, and chemical structure of these three samples were observed over 28 d. The bending curvature of NC-PNIPAm/Amidase decreased by 69% and 77% in its swelled and deswelled states, respectively, over the 28-d observation period (*SI Appendix*, Fig. S9 and Table S1). In contrast, diffusion of the mutant strain MM6 into NC-PNIPAm resulted in less change in bending curvature, decreasing by 10% in its swelled state and increasing by 2% in its deswelled state (*SI Appendix*, Fig. S9). Over 28 d NC-PNIPAm/BG-11(Km) exhibited a decrease in curvature of 5% and 17% in swelled and deswelled states, respectively (*SI Appendix*, Fig. S9).

The local Young’s modulus of NC-PNIPAm/Amidase decreased in both swelled and deswelled states over 28 d, by 80% and 75%, respectively (Fig. 5 *C* and *D* and *SI Appendix*, Table S4). In contrast, the local Young’s modulus for NC-PNIPAm/MM6 decreased by 30% in its swelled state and increased by 8% in its deswelled state over 28 d (Fig. 5 *C* and *D*). The observed changes in the local Young’s modulus of NC-PNIPAm/MM6 closely mimicked the cell-free sample, NC-PNIPAm/BG-11(7.7). However, the measurements for the local Young’s modulus values of NC-PNIPAm/MM6 in both the swelled and deswelled states were slightly lower than those of NC-PNIPAm/BG-11(7.7). These lower values could be caused by kanamycin in the growth medium used to culture strain MM6, as previous studies have shown that antibiotics can impact the modulus of hydrogel scaffolds and polymers (61). The local Young’s modulus of NC-PNIPAm/BG-11(Km) decreased by 41% in its swelled state and increased by 36% in its deswelled state over 28 d (Fig. 5 *C* and *D*). Therefore, it is likely that kanamycin (5 µg/mL) impacted the local Young’s modulus of swelled and deswelled NC-PNIPAm, though not as dramatically as the putative amidase. Additionally, the linear swelling and deswelling ratios for both NC-PNIPAm/MM6 and NC-PNIPAm/Amidase remained relatively unchanged over 28 d of observation. The linear swelling ratio of NC-PNIPAm/Amidase, NC-PNIPAm/MM6, and NC-PNIPAm/BG-11(Km) slightly decreased by 3%, 4%, and 1%, respectively (*SI Appendix*, Fig. S6*A* and Table S3). The linear deswelling ratio of NC-PNIPAm/Amidase, NC-PNIPAm/MM6, and NC-PNIPAm/BG-11(Km) decreased by 1%, 3%, and 18%, respectively, over the same period (*SI Appendix*, Fig. S6*B* and Table S3).

Purification of recombinant Synpcc7942_1548 protein with a C-terminal hexahistidine tag from *E. coli* cells resulted in a sample with numerous bands as visualized by SDS-PAGE (*SI Appendix*, Fig. S10), indicating the presence of contaminants and/or degraded forms of the protein. Bands larger than the putative amidase (49.2 kDa with tag) could correspond to complexes formed by the recombinant protein and amidase-binding proteins native to the *E. coli* host cells, where they modulate the activity of native peptidoglycan amidases (60). To ensure the partial degradation observed in NC-PNIPAm/Amidase was primarily driven by the recombinant, putative amidase and not by extraneous proteins that were not removed by purification, a clarified lysate of the *E. coli* expression strain BL21(DE3) was diffused into NC-PNIPAm at a final concentration of 45.5 µg/mL (*SI Appendix*, *Preparation of E. coli Lysate for Diffusion into NC-PNIPAm*), the same concentration used when diffusing the purified putative amidase into NC-PNIPAm. The local Young’s modulus of the resulting sample, NC-PNIPAm/Lysate, increased by 5% and 11% over 28 d in the swelled and deswelled states, respectively (Fig. 5 *C* and *D*). Additionally, the bending curvature remained relatively stable over 28 d of observation, with a slight decrease of 3% in the swelled bending curvature and a slight increase of 3% in the deswelled bending curvature (*SI Appendix*, Fig. S9). The linear swelling and deswelling ratios remained stable, decreasing and increasing by 1%, respectively (*SI Appendix*, Fig. S6).

ATR-FTIR results from NC-PNIPAm/Amidase demonstrated the same OH-bend between wavenumbers 1,440 and 1,395 cm^−1^ as NC-PNIPAm/Se, while NC-PNIPAm/MM6, NC-PNIPAm/BG-11(Km), and NC-PNIPAm/Lysate lacked this characteristic peak and were similar to the cell-free sample that did not exhibit partial degradation, NC-PNIPAm/BG-11(7.7) (Fig. 5 *E* and *F*). Thus, we specifically attribute the decrease in the local Young’s modulus previously observed in NC-PNIPAm/Se and NC-PNIPAm/Amidase to the presence of the *S. elongatus* putative amidase, in either the wild-type or recombinant forms.

**Fig. 6.**
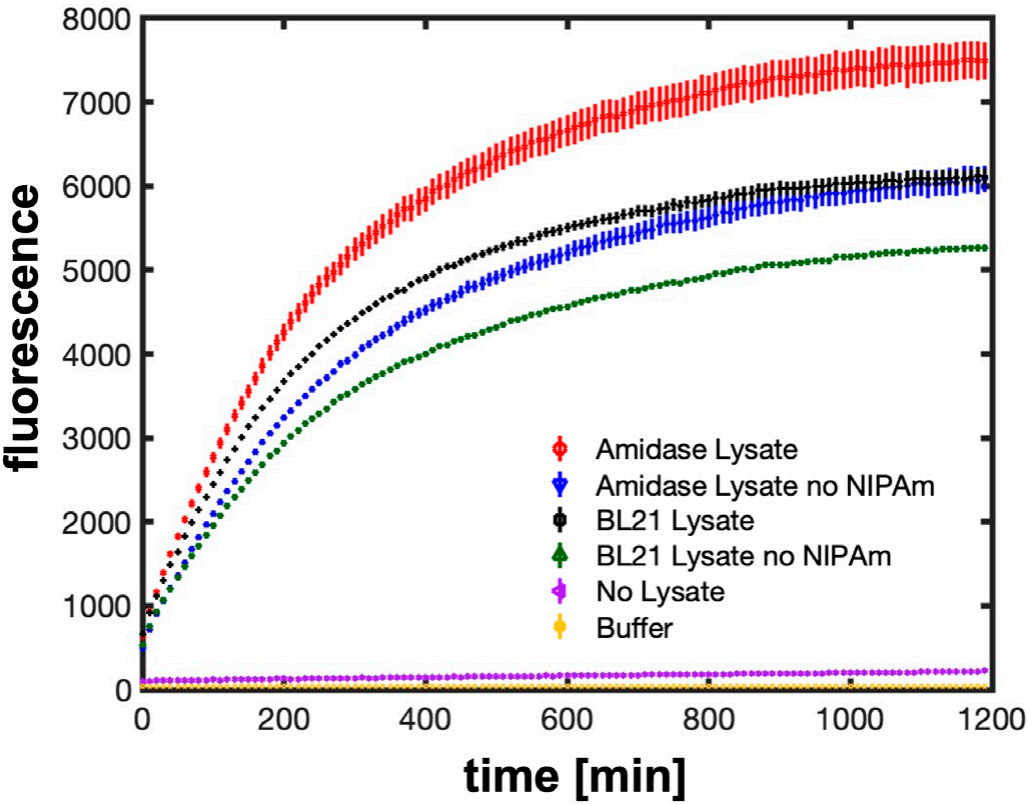
Detection of amidase activity. Fluorescence (excitation 485 nm; emission 538 nm) was monitored every 10 min for 20 h to detect the yellow dye formed by amidase activity in the presence of 4-Chloro-7-nitrobenzofurazan (NBD-Cl; *Materials and Methods*). A reaction containing NBD-Cl, NIPAm substrate, and a clarified lysate from *E. coli* cells expressing Synpcc7942_1548 is shown in red (“Amidase Lysate”). A similar reaction, omitting the NIPAm substrate, is shown in blue (“Amidase Lysate no NIPAm”). The reaction of NBD-Cl, substrate, and a clarified lysate of BL21 *E. coli* cells is shown in black (“BL21 Lysate”), and a similar reaction omitting NIPAm substrate is shown in green (“BL21 Lysate no NIPAm”). The purple dots correspond to a reaction in which the lysate component was substituted for buffer (“No lysate”), and golden dots show the negligible background fluorescence detected from 50 mM potassium phosphate buffer (pH = 7.5; “Buffer”). Each dot represents the average of all readings collected at a particular time point for that sample, and the bars indicate SE. The red, blue, black, and green dots are each the average of nine fluorescence values: triplicate reactions using material from distinct *E. coli* colonies that were plated in triplicate and measured over time. The purple and golden dots are each the average of three fluorescence measurements of a single reaction plated in triplicate.

To characterize the activity of the putative amidase from *S. elongatus*, two strains of *E. coli*, with and without expression of Synpcc7942_1548, were compared for their ability to generate a yellow dye by hydrolysis of NIPAm in the presence of 4-Chloro-7-nitrobenzofurazan (NBD-Cl) (62). Fluorescence (485 nm excitation, 538 nm emission) was monitored every ten minutes for 20 h. The assay background was high, demonstrating amidase activity in the lysate without either the *S. elongatus* putative amidase or NIPAm present, but compared to all other samples the lysate containing both the *S. elongatus* amidase and NIPAm produced significantly more fluorescence over time (Fig. 6). Thus, in combination with our previous observations, we conclude that the product of Synpcc7942_1548 is a secreted amidase with the ability to hydrolyze the amide bonds in NIPAm and NC-PNIPAm. The insertional mutant MM6, in which the amidase gene is inactivated, is therefore a potential candidate for continued efforts to use both NC-PNIPAm and *S. elongatus* in ELMs when a decrease in local Young’s modulus is not desired.

## Discussion

In this study, we show that *S. elongatus* cells can be introduced into temperature-responsive NC-PNIPAm hydrogels via a diffusion mechanism by exploiting its LCST. The *S. elongatus* cells show a gradient of growth within the NC-PNIPAm matrix, with a higher density of cells closer to the surface of the NC-PNIPAm, which creates a pseudo-bilayer within the NC-PNIPAm/Se ELM. This pseudo-bilayer causes a local Young’s modulus mismatch, resulting in a change in bending curvature in both its swelled and deswelled states. An observed decrease in the local Young’s modulus over time was the result of partial degradation of NC-PNIPAm primarily caused by a single enzyme, an amidase secreted by *S. elongatus* and encoded by the Synpcc7942_1548 locus. This amidase is designated AmiX, corresponding to the gene *amiX*. Our characterization of AmiX, and an insertional mutant strain incapable of producing it, establishes its role as an extracellular *S. elongatus* amidase capable of partially degrading NC-PNIPAm.

Diffusion mechanisms can enable the addition of microorganisms and other sensitive biological material into biocompatible hydrogels that are polymerized from cytotoxic monomer precursors. The proposed diffusion mechanism that we demonstrated was feasible due to the size of NC-PNIPAm pores being much larger than *S. elongatus* cells, on average, and due to the temperature-dependent shape-morphing capability of NC-PNIPAm (*SI Appendix*, Fig. S3). A more generalizable diffusion method was also attempted, which could be applied to hydrogels prepared with toxic monomers that are not inherently temperature responsive such as polyacrylamide (63) and polyacrylic acid (64), by dehydrating the gel then rehydrating it in cell culture. However, compared to our proposed diffusion mechanism, this method was much less efficient in terms of cell diffusion and subsequent proliferation within NC-PNIPAm, likely due to irreversible changes in porosity caused by complete dehydration (39). It is possible that partial dehydration mimicking the deswelling of NC-PNIPAm would mitigate changes in the microstructure and improve cell infiltration and proliferation after rehydration in cell culture, but this was not attempted here. Although this study has demonstrated the utility of a diffusion mechanism in the creation of NC-PNIPAm/Se, additional combinations of microorganisms, substrates with varying pore sizes, and dehydration conditions would need to be tested to confirm the broad utility of diffusion-based mechanisms for preparing ELMs.

Further studies could also seek to address the function of AmiX in *S. elongatus*. One possibility is that AmiX is utilized for nitrogen acquisition; unlike some cyanobacteria such as the Nostocales, *S. elongatus* is unable to fix dinitrogen and instead depends on sources of combined nitrogen. A secreted enzyme hydrolyzing amide bonds in compounds surrounding the cells could create a pool of combined nitrogen sources for *S. elongatus* to utilize. The optimal enzyme for this scenario would be active under a broad range of conditions (such as pH 7.7-10.0, as described in this study) and hydrolyze a variety of substrates. These assumptions could be tested by analyzing the specificity of recombinant AmiX, as well as the effect of AmiX activity on nitrogen-starved *S. elongatus* cells. Amidases have also been identified in the exoproteome of the filamentous cyanobacterium *Anabaena* sp. PCC 7120, which uses them to create nanopores in peptidoglycan at septal junctions, facilitating cell-to-cell signaling (65). Although this study has identified and characterized AmiX in the context of NC-PNIPAm/Se, further questions remain regarding the role of this extracellular enzyme in *S. elongatus* and other cyanobacteria bearing the *amiX* gene.

By combining the photosynthetic cyanobacterium *S. elongatus* with the temperature-responsive material NC-PNIPAm, we showcased a diffusion-based mechanism for ELM preparation. Observation of this material in nonequilibrium conditions also led to the identification and characterization of an enzyme that irreversibly altered the properties of the ELM over time. It is our intention that this multifaceted approach to the creation and characterization of an ELM will spur the development of increasingly complex ELMs that can respond to multiple stimuli.

## Materials and Methods

N-isopropylacrylamide (NIPAm, stabilized with 4-methoxyphenol, M_W_ = 113.16 g/mol) was purchased from Tokyo Chemical Industry (TCI) America. N,N’-methylenebisacrylamide (BIS), 2-hydroxy-4′-(2-hydroxyethoxy)-2-methylpropiophenone (Irgacure 2959), and (tridecafluoro-1,1,2,2 tetrahydrooctyl) dimethylchlorosilane (silane) were purchased from Sigma-Aldrich (St. Louis, MO). Nanoclay (NC, Laponite-RD) was donated by BYK Additives & Instruments (Wessel, Germany). All chemicals were used as received without additional purification. Unless otherwise stated, *S. elongatus* PCC 7942 (*S. elongatus*) strain AMC06 (66) from the Golden laboratory was used in the creation of all ELMs.

### Preparation of Synpcc7942_1548 Insertional Mutant Strain MM6.

The insertional mutant in the Synpcc7942_1548 locus of *S. elongatus*, named strain MM6, was created by transformation and homologous recombination of plasmid p8S1-MM6 into the *S. elongatus* genome, replacing the native locus with the interrupted variant. Plasmid p8S1-MM6 was originally created as one of 2,451 plasmid constructs in a library for generating insertional mutants in every nonessential gene of *S. elongatus* (67, 68) and was stored frozen in *E. coli* host cells in the Golden laboratory. The host *E. coli* carrying p8S1-MM6 was revived in Luria-Bertani (LB) broth containing 50 µg·mL^−1^ kanamycin by shaking overnight at 250 RPM, 37 °C, and plasmid DNA was extracted from the culture using the QIAPrep Spin Miniprep Kit (Qiagen). In plasmid p8S1-MM6, the Synpcc7942_1548 open reading frame is interrupted at the 342nd base by a 2,001 base pair (bp) insertion encoding kanamycin resistance (67, 68). The plasmid also contains flanking regions homologous to the Synpcc7942_1548 locus in *S. elongatus* to facilitate recombination into the chromosome. They span 1,756 bp upstream of the insertion site and 8,688 bp downstream. Plasmid p8S1-MM6 was used to transform *S. elongatus,* and a segregated mutant was verified as described in *SI Appendix*, *Transformation of S. elongatus*.

### Colorimetric Amidase Assay.

As demonstrated by Henke and Bornscheuer, 4-Chloro-7-nitrobenzofurazan (NBD-Cl) reacts with amines, such as those resulting from amide hydrolysis by an amidase, to form a yellow dye in aqueous solution at room temperature (62). A total of thirteen reactions were prepared to detect amidase activity in this manner. Six reactions were prepared by adding 100 µL of each lysate (three replicate lysates from BL21 cells expressing Synpcc7942_1548, and three replicate lysates from unaltered BL21) to six tubes containing 800 µL 50 mM potassium phosphate buffer (pH = 7.5), 50 µL 40 mM 4-Chloro-7-nitrobenzofurazan (NBD-Cl), and 50 µL of substrate (50 mM NIPAm). Another six reactions were prepared the same way, except with 50 µL of phosphate buffer instead of NIPAm substrate (the “no NIPAm” reactions). A thirteenth reaction was prepared by substituting lysate with 100 µL phosphate buffer and retaining all other components (the Buffer reaction). Preparation and subsequent pipette mixing was performed in dim light due to the sensitivity of NBD-Cl. 200 µL samples of each reaction were transferred in triplicate into wells of a clear-bottomed 96-well plate. Three additional wells were filled with phosphate buffer. The fluorescence of each well (485 nm excitation; 538 nm emission) was monitored every 10 min after shaking (3 s, 2.5 mm) for 20 h using a Tecan Infinite model M200 plate reader (Tecan, Switzerland) (Fig. 6).

### Preparation of NC-PNIPAm Precursor ink.

NIPAm solution (2.0 M) was prepared using DI water and mixed with a stir bar until the NIPAm was fully dissolved. BIS solution (0.13 M) was prepared using DI water and mixed by utilizing a vortex mixer until the BIS was fully dissolved. Once prepared, 10 mL of NIPAm monomer solution (2.0 M), 120 μL of BIS crosslinker solution (0.13 M), 0.04 g Irgacure 2959 photoinitiator, and 1.0 g NC rheological modifier were added into a 35 mL container (Thinky USA, Inc.) and mixed in 1-min increments until homogeneous. The homogenous ink was loaded into a 3 mL syringe and centrifuged at 2,600 rpm for 5 min to remove any air bubbles.

### Preparation of NC-PNIPAm/Se and other NC-PNIPAm Samples Via Diffusion.

To prepare the stimuli-responsive ELM, NC-PNIPAm/Se, *S. elongatus* cells were introduced into the UV crosslinked NC-PNIPAm hydrogel matrix via diffusion (Fig. 1). After crosslinking, the NC-PNIPAm structure was placed into a Petri dish filled with Milli-Q water at 22 °C for at least 2 h to fully swell. The swollen NC-PNIPAm structure was then transferred to a Petri dish filled with preheated Milli-Q water and kept at 37 °C for 2 h to deswell. The deswelled NC-PNIPAm structure was transferred to a Petri dish filled with *S. elongatus* culture (OD_750_ = 0.8) at 22 °C for at least 2 h to allow the structure to reswell, incorporating *S. elongatus* cells into the hydrogel matrix and creating the ELM NC-PNIPAm/Se (Fig. 1). NC-PNIPAm/Se was transferred to fresh BG-11 growth medium for storage and growth at room temperature.

The same diffusion mechanism was employed to prepare all other samples. Once deswelled, the NC-PNIPAm structure was transferred to a Petri dish filled with one of the following seven solutions: buffered BG-11 (pH = 7.7), buffered BG-11 (pH = 10.0), buffered *S. elongatus* conditioned medium (pH = 7.7), buffered *S. elongatus* conditioned medium (pH = 10.0), *S. elongatus* conditioned medium treated with protease and autoclaved, BG-11 (pH = 7.5) with purified Synpcc7942_1548 protein added to 45.5 µg·mL^−1^, or medium conditioned by the growth of strain MM6. Once reswelled in their respective solution, the NC-PNIPAm samples were transferred to new petri dishes containing fresh BG-11 medium buffered to its respective pH for storage at room temperature.

### Fabrication of Casted Structures.

The casting approach was used to synthesize uniform, flat, and thin NC-PNIPAm structures for characterization testing. The NC-PNIPAm precursor ink was loaded in a 3 mL syringe, centrifuged at 2,600 rpm for 5 min to remove bubbles, and injected between two clean glass slides (75 mm × 25 mm × 1 mm) separated by two pieces of Kapton film placed on either edge. 25 µm-thick Kapton film was used in the preparation of samples for characterizing the diffusion coefficient of *S. elongatus* cells through the NC-PNIPAm hydrogel matrix and the hydrogel curvature. 100 µm-thick Kapton film was used in the preparation of samples for the following measurements: *S. elongatus* cell viability in NC-PNIPAm via confocal fluorescence microscopy; mechanical properties of ELMs and cell-free samples; Fourier Transform Infrared Spectroscopy (FTIR) of ELMs and cell-free samples; and the linear swelling and deswelling ratios of ELMs and cell-free samples. Once injected, the cast structure was cured using UV irradiation (Omnicure S2000, Excelitas Technologies) at 60 mW·cm^−2^ for 2 min before undergoing the diffusion process.

### ELM and Hydrogel Sample Storage Conditions.

Following the introduction of cells or conditioned media, all ELM and NC-PNIPAm samples in this study were stored in sterile Petri dishes (35 mm diameter and 10 mm height) with sufficient BG-11 growth medium (~5 mL) to cover the entire surface. Additional medium was added before evaporation exposed the ELM surface to air. The Petri dishes were kept on the benchtop at 25 °C and received ~40 µmol photons·m^−2^·s^−1^ light. The media surrounding ELM samples gradually became green as escaped cells grew. Before microscopy, ELM samples were washed by replacing the surrounding media three times with fresh BG-11 to remove escaped cells.

### Viability of *S. elongatus* Cells Within ELM Via Confocal Fluorescence Microscopy.

Discs (8.95 mm diameter and 0.1 mm thickness) were punched from cast sheets of NC-PNIPAm/Se and placed in a 35 mm glass-bottom Petri dish (Matsunami Glass Ind, LTD). ELM discs were imaged using confocal fluorescence microscopy (Leica Sp8 STED with Falcon) immediately following fabrication and after 28 d of growth to evaluate the viability of *S. elongatus* cells within NC-PNIPAm (Fig. 2). The samples were imaged using the Tetramethylrhodamine (TRITC) filter (excitation 555/528 nm and emission 617/673 nm) to visualize the autofluorescence of *S. elongatus* cells. Images were then reconstructed in 3-D using Arivis Vision4D software (Fig. 2).

### Experimental Diffusion Coefficient of *S. elongatus* Cells Entering the NC-PNIPAm Hydrogel Matrix.

To characterize the diffusion coefficient of *S. elongatus* cells entering the NC-PNIPAm hydrogel matrix, a 1-D infinite diffusion set up was assembled (Fig. 3 *A* and *B*). Rectangular sheets of NC-PNIPAm (20 mm × 5 mm × 0.25 mm) were deswelled in a 40 °C bath of Milli-Q water for 1 h. A deswelled sample was patted dry with a KimWipe and transferred to a glass microscope slide (75 mm × 25 mm × 1 mm) and placed on the microscope (NIKON, H600L) and 5 µL *S. elongatus* culture (OD_750_ = 0.3) was pipetted onto its surface. Immediately after pipetting, the microscope was focused just below the material surface and images were captured of the *S. elongatus* cells diffusing into the NC-PNIPAm every 5 s for 5 min. These images were analyzed to track the movement of *S. elongatus* cells into the hydrogel using the open sourced TrackMate plugin for FIJI as previously demonstrated by Ershov and coworkers (43, 45, 46). Cell trajectories were then exported and analyzed using the open source msdanalyzer package in MATLAB as described by Tarantino and coworkers (Fig. 3) (46). Three trials were conducted and averaged to obtain the experimental diffusion coefficient. This method of diffusion was only utilized in the data pertaining to Fig. 3, all other instances where diffusion was applied, see *SI Appendix*, *Preparation of NC-PNIPAm/Se and Other NC-PNIPAm Samples via Diffusion*.

### Theoretical Diffusion Coefficient of *S. elongatus* Cells Entering the NC-PNIPAm Hydrogel Matrix.

The theoretical hydrodynamic radius (*R_H_*) was calculated to account for rod-shaped particles using the following equation where *λ* represents the aspect ratio of the rod-shaped particle studied by Ortega and de la Torre (47).

$$\begin{array}{c} R_{H}={\left(\frac{3}{2}\lambda \right)}^{\frac{1}{3}}\;\;R\left[1.009+1.395*10^{-12}\left(\ln\lambda \right)+7.88*10^{-2}{\left(\ln\lambda \right)}^{2}+6.04*10^{-3}{\left(\ln\lambda \right)}^{3}\right]. \end{array}$$

The resulting theoretical hydrodynamic radius of each *S. elongatus* cell was 3.1 µm and was utilized to calculate the theoretical diffusion coefficient with the Stokes–Einstein equation derived for two-dimensions where *k_B_* represents the Boltzmann constant, *T* represents temperature, *η* represents the kinematic viscosity of the solvent, and *R_H_* represents the hydrodynamic radius of rod-shaped particles (48).

$$D_{t}=k_{B}T/4\pi \eta R_{H}.$$

### Characterization.

Bending curvature measurements were obtained by measuring the radius of curvature of each ELM and cell-free NC-PNIPAm sample (10 mm × 2.5 mm × 0.25 mm) using the radius measuring function on an optical microscope (VHX1000, Keyence, IL). Samples for curvature measurements were all sliced in the same orientation relative to the shear direction. The curvature was calculated using *K* = *1/R*, where *K* represents bending curvature and *R* represents the measured radius of curvature (Fig. 4 *A* and *B*). Local Young’s modulus measurements of ELM and cell-free NC-PNIPAm samples (8.95 mm diameter × 0.1 mm thickness) were acquired using a nanoindenter (Piuma, Optics 11 Life, Netherlands) and probe with a stiffness of 0.5 N·m^−1^ and spherical tip radius of 50 µm. Bending curvature and local Young’s modulus measurements were taken for all samples in both swelled and deswelled states. Linear swelling and deswelling ratio measurements were acquired by using the diameter measuring function on an optical microscope. The linear swelling ratio (*λ*_s_) was calculated using the equation $\lambda_{s}=D_{s}/D_{0}$, where *D_s_* represents the diameter of a fully swelled sample and *D_0_* represents the diameter of the as-prepared sample. The linear deswelling ratio (*λ*_d_) was calculated using the equation $\lambda_{d}=D_{d}/D_{0}$, where *D_d_* represents the diameter of a fully deswelled sample and *D_0_* represents the diameter of the as-prepared sample. For measurements taken in the deswelled state, samples were preheated for 2 h prior to testing and continuously heated with a heating stage (Precision Temperature Controller, Biosciences Tools, CA). All characterization measurements were periodically acquired immediately after fabrication until the 28th day of storage (Fig. 4).

### Attenuated Total Reflection–FTIS.

On the 28th day of observation, the partial degradation of NC-PNIPAm/Se and cell-free NC-PNIPAm samples was assessed using Attenuated Total Reflection-FTIS (ATR-FTIR) (Nicolet iS50 and 6700, Thermo Fisher). Small discs (8.95 mm diameter and 0.1 mm thickness) were punched out of NC-PNIPAm/Se and cell-free NC-PNIPAm samples and frozen at −80 °C for 24 h. Once frozen, the samples were transferred to a lyophilizer (FreeZone, Labconco, KS) at 22 °C for 12 h. After freeze-drying, the samples were tested using ATR-FTIR with 32 scans and a resolution of 4 (Fig. 5).

## Supplementary Material

Appendix 01 (PDF)

Movie S1. ***S. elongatus* diffusion into NC-PNIPAm.** Time lapsed video of *S. elongatus* cell diffusion into thin NC-PNIPAm sheet (20 mm x 5 mm x 0.25 mm). Video is comprised of sequential images captured every 5 seconds over 5 minutes using fluorescence microscopy. Time lapse images were stitched together are played at 25X speed.

## Data Availability

All study data are included in the article and/or supporting information.
