# Supplementary material for: A responsive living material prepared by diffusion reveals extracellular enzyme activity of cyanobacteria
Source: Proc Natl Acad Sci U S A. 2025 May 1;122(18):e2424405122. doi: 10.1073/pnas.2424405122 (PMC12067278; doi:10.1073/pnas.2424405122)
Supplement: Supplementary file 1 — Appendix 01 (PDF) [file pnas.2424405122.sapp.pdf]

PNAS

Supporting Information for
A responsive living material prepared by diffusion reveals extracellular enzyme activity of cyanobacteria

Lisa Tang[1†], Nathan Soulier[2†], Rebecca Wheeler[1], Jonathan K. Pokorski[1], James W. Golden[2], Susan S. Golden[2*], Jinhye Bae[1,3,4*]

*Corresponding authors: Jinhye Bae and Susan S. Golden
Email: j3bae@ucsd.edu and sgolden@ucsd.edu

This PDF file includes:

Supporting Materials and Methods
Fig. S1. Fig. S1. Viability of *S. elongatus* cells observed through fluorescence microscopy and macroscopic imaging.
Fig. S2. Alternative diffusion methods.
Fig. S3. Scanning electron microscopy.
Fig. S4. Testing lower critical solution temperature (LCST) through heating and cooling cycles.
Fig. S5. Temperature-dependent bending curvature changes over 28 days in 100 μm thick NC-PNIPAm/Se compared to 100 μm thick NC-PNIPAm/BG-11(7.7).
Fig. S6. Changes in the swelling and de-swelling ratios of NC-PNIPAm in ten conditions over 28 days.
Fig. S7. Changes in local Young's modulus and bending curvature of NC-PNIPAm/CM(7.7), NC-PNIPAm/CM(10), NC-PNIPAm/CM(Aclv), and NC-PNIPAm/BG-11(10) over 28 days.
Fig. S8. ATR-FTIR spectra of NC-PNIPAm/BG-11(7.7), NC-PNIPAm/Se, NC-PNIPAm/CM(7.7), NC-PNIPAm/CM(10), NC-PNIPAm/CM(Aclv), and NC-PNIPAm/BG-11(10).
Fig. S9. Monitoring the bending curvature of NC-PNIPAm/BG-11(Km), NC-PNIPAm/MM6, NC-PNIPAm/Amidase, and NC-PNIPAm/Lysate over 28 days.
Fig. S10. SDS-PAGE gel visualizing the recombinant putative amidase Synpcc7942_1548 after purification from lysed *E. coli* cells by immobilized metal affinity chromatography.
Table S1. Bending curvature of 25 μm thick NC-PNIPAm incorporated with different cells or solutions.
Table S2. Abbreviation definitions and their respective material compositions.
Table S3. Linear swelling and de-swelling ratios of NC-PNIPAm incorporated with different cells or solutions.
Table S4. Local Young's modulus of NC-PNIPAm incorporated with different cells or solutions.
Table S5. DNA primers used in this study with restriction sites in capital letters.
Table S6. Plasmids used in this study.

Other supporting materials for this manuscript include the following:

Movie S1 (.mp4 format). *S. elongatus* diffusion into NC-PNIPAm.

Supporting Information Text

Supporting Materials and Methods

Preparation of cell growth medium

Blue green 11 (BG-11) medium is an optimized solution for *S. elongatus* growth. The following solutions were added to 983 mL of deionized (DI) water in a 1 L bottle for a final volume of 1 L: 10 mL of blue green 1 (BG-1) and 1 mL each of stock solutions BG-2 through BG-8.

The BG-1 through BG-8 stock solutions were prepared as follows: BG-1, sodium nitrate (150 $g{\cdot}L^{-1}$); BG-2, calcium chloride, dihydrate (36 $g{\cdot}L^{-1}$); BG-3, ferric ammonium citrate (6 $g{\cdot}L^{-1}$) and citric acid (6 $g{\cdot}L^{-1}$); BG-4, disodium ethylenediaminetetraacetic acid (EDTA; 1 $g{\cdot}L^{-1}$); BG-5, potassium phosphate, dibasic (40 $g{\cdot}L^{-1}$); BG-6, magnesium sulfate, heptahydrate (75 $g{\cdot}L^{-1}$); BG-7, sodium carbonate (20 $g{\cdot}L^{-1}$); BG-8, Boric acid (2.83 $g{\cdot}L^{-1}$), Manganese (II) chloride, tetrahydrate (1.81 $g{\cdot}L^{-1}$), Zinc sulfate, heptahydrate (0.222 $g{\cdot}L^{-1}$), Sodium molybdate, dihydrate (0.39 $g{\cdot}L^{-1}$), Copper (II) sulfate, pentahydrate (0.079 $g{\cdot}L^{-1}$), and Cobalt (II) nitrate, hexahydrate (0.049 $g{\cdot}L^{-1}$).

Normally BG-11 was adjusted to pH=7.5 with hydrochloric acid (HCl) prior to autoclaving. For the buffered BG-11 media, two buffering solutions were prepared. One was a solution of 1.0 M tris(hydroxymethyl)aminomethane (Tris)-HCl prepared by dissolving 24.2 g Tris in 150 mL DI water, adjusting the pH to 8 using 5.0 M HCl and a pH meter (APERA Instruments, PH700), and adding DI water to 200 mL. The second buffering solution, 0.8 M glycine (pH=10.0), was prepared by dissolving 6 g glycine in 80 mL DI water, which was adjusted to pH=10.0 using NaOH (5 M) prior to addition of DI water to 100 mL. For buffered BG-11 pH=7.7, the 1.0 M (Tris)-HCl (pH=8.0) solution was added to a final concentration of 10 mM, followed by adjustment to pH=7.7 with 1.0 M hydrochloric acid (HCl). Buffered BG-11 pH=10.0 was prepared by adding the 0.8 M glycine solution (pH=10.0) to 10 mM.

Preparation of conditioned media

A culture of *S. elongatus* (100 mL) was grown for approximately three weeks at 30°C under ~100 μmol photons$\cdot m^{-2}\cdot s^{-1}$ light with shaking (120 RPM) to an optical density (at 750 nm; OD_{750}) of 1.5 and the culture was transferred to sterile 50 mL conical tubes and centrifuged for 30 minutes at 4600×*g*. To remove cells, the medium was decanted from the green cell pellet and filtered using a vacuum filtration cartridge containing a polyether sulfone (PES) membrane (pore-size 0.2 μm, Thermo Fisher).

A 15 mL portion of the filtered conditioned medium was adjusted to pH=7.7 by adding 1.0 M Tris-HCl (pH=8.0) to 10 mM, followed by adjustment to pH=7.7 with 1.0 M hydrochloric acid (HCl). Another 15 mL portion was adjusted to pH=10.0 by adding 0.8 M Glycine-NaOH (pH=10.0) to 10 mM. Proteinase K and calcium chloride were added to 0.1 mg/mL and 5 mM in 50 mL of the remaining conditioned medium, respectively, and incubated at 37°C for 24 hours prior to autoclaving for 45 minutes at 121°C. The pH=7.7, pH=10.0, and autoclaved conditioned media were stored at 4°C for 24 hours prior to use.

Transformation of *S. elongatus*

S. elongatus was cultured by inoculating 100 mL of BG-11 in an autoclaved 200 mL conical flask with cells scraped from a BG-11 agar plate. The inoculated culture was shaken at 120 rpm (MaxQ 2000 Orbital Shaker, Thermo Fisher) at 30°C under 174–199 μmol photons$\cdot m^{-2}\cdot s^{-1}$ light. Cells to be used for transformation were grown for three days (OD_{750} = 0.5) prior to transfer to a shaker-incubator capable of light-dark cycling (Geneva Scientific model I-22LLX), where the culture was subjected to 12 hours of light (100 μmol photons$\cdot m^{-2}\cdot s^{-1}$) and 12 hours of darkness for three-and-a-half days. One hour into the dark period on the fourth day, 5 mL of cell culture was collected, pelleted by centrifugation (5 minutes, 4600×*g*), and resuspended in 1 mL of fresh BG-11 growth medium. Centrifugation and resuspension were repeated once prior to addition of 500 ng plasmid

(p8S1-MM6) DNA. The resuspended cells and plasmid DNA were mixed by pipette and allowed to incubate in darkness at 30°C for fourteen hours.

After incubation, cells were pelleted by centrifugation (5 minutes at 4600×*g*) and resuspended in 100 µL of fresh BG-11 medium. The resuspended cells were then spread on a BG-11 agar plate containing 5 µg·mL^{-1} kanamycin. The plate was incubated at 30°C in 150 µmol photons·m^{-2}·s^{-1} light for ten days, then colonies were picked and streaked in small patches on a fresh BG-11 plate made with 5 µg·mL^{-1} kanamycin, to maintain selection for mutant chromosomes. After four days, cell material from each patch was once more streaked on a fresh antibiotic plate containing 5 µg·mL^{-1} kanamycin and grown under the same conditions. After another three days, patches of cells from the second patch-plate were screened for complete segregation of the mutant genotype by PCR and Sanger sequencing of the Synpcc7942_1548 locus using primers “1548 F” and “1548 R” **(Fig. 5B; Supporting Table 5)**. Cells scraped from a segregated patch were used to inoculate 100 mL of BG-11 in an autoclaved 250 mL conical flask containing 5 µg·mL^{-1} kanamycin to produce a working culture of the mutant strain.

Preparation of purified amidase

The Synpcc7942_1548 gene was PCR amplified to encode a C-terminal hexa-histidine tag using the “Am2991 F” and “Am6H2991 R” primer set **(Supporting Table 6)**. This amplicon contained flanking regions (~30 bp) corresponding to either side of the EcoRI digestion site in plasmid pAM2991(1). Plasmid pAM2991 was digested with EcoRI prior to insertion of the amplicon by Gibson assembly(2), resulting in plasmid pAmidase6H **(Supporting Table 6)**. After incubation at 50°C for an hour, the entirety of the Gibson assembly reaction (20 µL) was added to a tube containing 50 µL of chemically competent *E. coli* cells (DH5α) frozen in 10% glycerol, and allowed to thaw on ice. Once thawed, the cell-DNA solution was mixed by pipette and incubated on ice for ten minutes, followed by heat-shock treatment for one minute at 42°C. The tube was immediately transferred to ice for two minutes prior to addition of 400 µL fresh LB broth. Cells were then allowed to recover for 1 hour at 37°C with gentle rotation. Cells were then pelleted by centrifugation (1 minute at 9400×*g*) prior to resuspension in 100 µL LB broth and plating on an LB-agar plate containing both streptomycin (Sp; 20 µg·mL^{-1}) and spectinomycin (Sm; 20 µg·mL^{-1}).

After fourteen hours at 37°C, colonies on the plate were assayed for the presence of the Synpcc7942_1548 gene by colony PCR using primers “1548 F” and “1548 R” **(Supporting Table 5)**. Three PCR-verified colonies were picked and introduced into individual glass tubes containing 10 mL LB broth and 20 µg·mL^{-1} Sp/Sm. The tubes were capped and shaken at 37°C, 300 RPM for ten hours prior to extraction of plasmid DNA using the QIAPrep Spin Miniprep Kit. The sequence of the recombinant Synpcc7942_1548 gene in each preparation of pAmidase6H was verified by Sanger sequencing using primers “pTRC Fwd” and “2991 CSRev” (**Supporting Table 5)**. Verified plasmid was used to transform chemically competent *E. coli* BL21(DE3) cells by heat shock (30 seconds at 37°C) before recovery for 2 minutes on ice and plating on LB-agar plates containing 20 µg·mL^{-1} Sp/Sm. After fourteen hours at 37°C, colonies were assayed for the presence of the putative amidase gene by PCR using primers “pTRC Fwd” and “2991 CSRev” (**Supporting Table 5)**. Three starter cultures were made by introducing a PCR-positive colony into 75 mL LB broth containing 20 µg·mL^{-1} Sp/Sm. The starter cultures were grown at 37°C for twelve hours with shaking (300 RPM) then diluted into 1 L LB broth containing 20 µg·mL^{-1} Sp/Sm. These 1 L cultures were grown at 37°C for two hours with shaking (300 RPM) prior to the addition of isopropyl β-D-1-thiogalactopyranoside (IPTG) to 1 mM. IPTG-induced cultures were allowed to grow for another six hours at 30°C prior to harvesting cells by centrifugation at 9400×*g*, 4°C for 30 minutes. The supernatant was removed and cell pellets were stored at -20°C until use. Pellets were thawed at room temperature, resuspended in 20 mM TRIS (pH=8.0) and 20 mM NaCl (Buffer20), and pooled prior to lysis with a High-Pressure Homogenizer (Emulsiflex C-3; Avestin, Ottawa, Canada). The resulting cell lysate was clarified by centrifugation (9400×*g* for 30 minutes at 4°C) prior to being added to a column packed with nickel-NTA resin and equilibrated with Buffer20. After passage of the entire lysate through the column bed twice, the resin was washed with three column volumes of Buffer20, followed by three column volumes of 20 mM TRIS (pH=8.0) + 200 mM NaCl. The resin was again washed with three column volumes of Buffer20 before washing with three column volumes of Buffer20 + 20 mM imidazole. The final elution was carried out in Buffer20 + 500 mM

Imidazole. Centrifugal filter cartridges (Amicon Ultra 0.5, 10k MWCO, Millipore Sigma) were used to perform buffer exchange of the purified sample into Buffer20, and the final protein concentration of 20 mg·mL^{-1} was determined by Bradford assay (Coomassie Plus, Thermo Fisher). Samples of purified putative amidase were analyzed by SDS-PAGE **(Supporting Fig. 9)**. The purified sample was added to a final concentration of 45.5 µg·mL^{-1} in 100 mL BG-11 for diffusion into NC-PNIPAm.

Preparation of *E. coli* lysate for diffusion into NC-PNIPAm
A colony of BL21(DE3) was used to inoculate a flask containing 75 mL LB broth. After shaking (300 RPM) for twelve hours at 37°C, the 75 mL culture was used to inoculate a flask containing 1 L LB broth. The 1 L culture was shaken (300 RPM) for six hours at 37°C prior to harvesting the cells by centrifugation (9400×*g* for 30 minutes at 4°C). The LB was removed and cell pellets were stored at -20°C until use. Pellets were thawed at room temperature, resuspended in Buffer20, and pooled for lysis as described above. The resulting lysate was clarified by centrifugation (9400×*g* for 30 minutes at 4°C), and total protein concentration (40.0 mg·mL^{-1}) was determined by Bradford. The lysate was added to a final concentration of 45.5 µg·mL^{-1} in 100 mL BG-11 for diffusion into NC-PNIPAm.

Preparation of lysates for amidase assay
Three cultures were prepared by inoculating 75 mL LB broth containing 20 µg·mL^{-1} Sp/Sm with separate BL21(DE3) colonies verified for the presence of the Synpcc7942_1548 gene by PCR using primers "1548 F" and "1548 R" (**Supporting Table 5**). Three more cultures were prepared by inoculating 75 mL LB broth (no antibiotics) with BL21(DE3) colonies that had not been transformed with any expression constructs. After shaking (300 RPM) at 37°C for fourteen hours, each culture was independently pelleted in 50 mL tubes by two rounds of centrifugation at 7000×*g*, 4°C for 15 minutes. The supernatant was removed after each round of centrifugation for all samples, and the pellets were ultimately resuspended in 3 mL of 50 mM potassium phosphate buffer (pH=7.5). The cells in each sample were then lysed by sonication using six five-second pulses at 50% amplitude followed by twelve minutes on ice, repeated three times per sample (Sonic Dismembrator model 500, Thermo Fisher). The resulting lysates were clarified by centrifugation (10,000×*g* for 4 minutes at room temperature) and the supernatants were collected. All six clarified lysate samples, three originating from BL21(DE3) colonies expressing the Synpcc7942_1548 gene and three from BL21(DE3) colonies with no expression construct, were normalized to a total protein concentration of 2.0 mg·mL^{-1} determined by Bradford assays (Coomassie Plus, Thermo Fisher).

Fabrication of 3D printed structures
3D printed NC-PNIPAm structures were fabricated using a direct-ink-writing approach with a pneumatic 3D printer (BIO X, Cellink, Sweden). 3D printed structures were fabricated with low infill density and higher surface area to promote *S. elongatus* cell growth. A 30-gauge dispensing needle with a conical tip was attached to the 3 mL syringe loaded with the centrifuged NC-PNIPAm precursor ink. The NC-PNIPAm precursor ink was pneumatically extruded onto a glass slide (75 mm x 50 mm x 1 mm) at a speed of 4.0 mm·s^{-1} with a corresponding pressure of 65 kPa. Once printed, the structure was cured using UV irradiation (60 mW·cm^{-2} for 2 minutes) prior to undergoing the diffusion process.

Scanning Electron Microscopy Imaging
NC-PNIPAm/BG-11 as prepared by diffusion was assessed through Scanning Electron Microscopy (SEM) (Phenom G6 Pro SEM, Thermo Fisher) imaging. Small discs (8.95 mm diameter and 0.1 mm thickness) of NC-PNIPAm/BG-11 were biopsied and frozen using liquid nitrogen (Airgas, KS). Once frozen, the samples were moved to the lyophilizer (FreeZone, Labconco, KS) at 22°C for a minimum of 24 hours. After freeze drying, samples were split down the center to expose the cross section of the NC-PNIPAm/BG-11 and imaged on an SEM using a backscattered electron detector (BSD) with 5 kV.

Preparation of silane treated plastic petri dishes and glass coverslips

Plastic Petri dishes and glass coverslips used in storage and observation of hydrogel curvature were treated with silane to minimize adhesion of hydrogel samples to surfaces, ensuring that all bending curvature measurements of NC-PNIPAm were acquired from free-standing samples. Untreated plastic Petri dishes (35 mm diameter and 10 mm height) and glass coverslips (22 mm x 22 mm x 0.15 mm) were first cleaned with isopropyl alcohol and gently dried using an air blower. The cleaned Petri dishes and glass coverslips were transferred to a desiccator. Two drops of (Tridecafluoro-1,1,2,2 Tetrahydrooctyl) dimethylchlorosilane (silane) were pipetted onto a single glass slide, which serves as a reservoir. The desiccator was then vacuumed using a pump overnight. The silane-treated Petri dishes and glass coverslips were removed from the desiccator and cleaned with isopropyl alcohol, then stored in a parafilm-sealed large Petri dish (150 mm diameter and 15 mm height) until use.

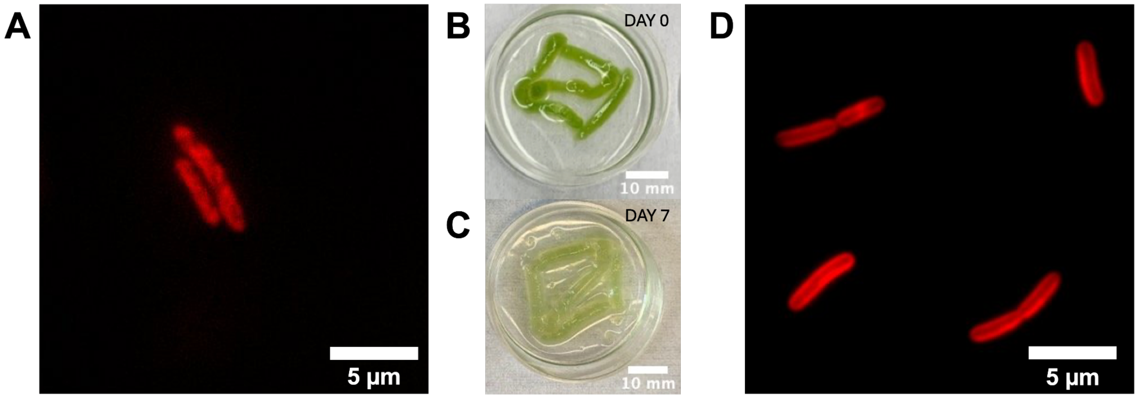

Fig. S1. Viability of *S. elongatus* cells observed through fluorescence microscopy and macroscopic imaging. **(A)** Fluorescent microscope image of *S. elongatus* cells when exposed to NIPAm monomer in the NC-PNIPAm precursor solution during the one-pot mixing process showing an uneven appearance of autofluorescence of *S. elongatus* cells. Macroscopic images of NC-PNIPAm/Se as prepared through one-pot mixing taken on **(B)** Day 0 and **(C)** Day 7. **(D)** Fluorescent microscope image of healthy *S. elongatus* cells immobilized on the surface of agar plates.

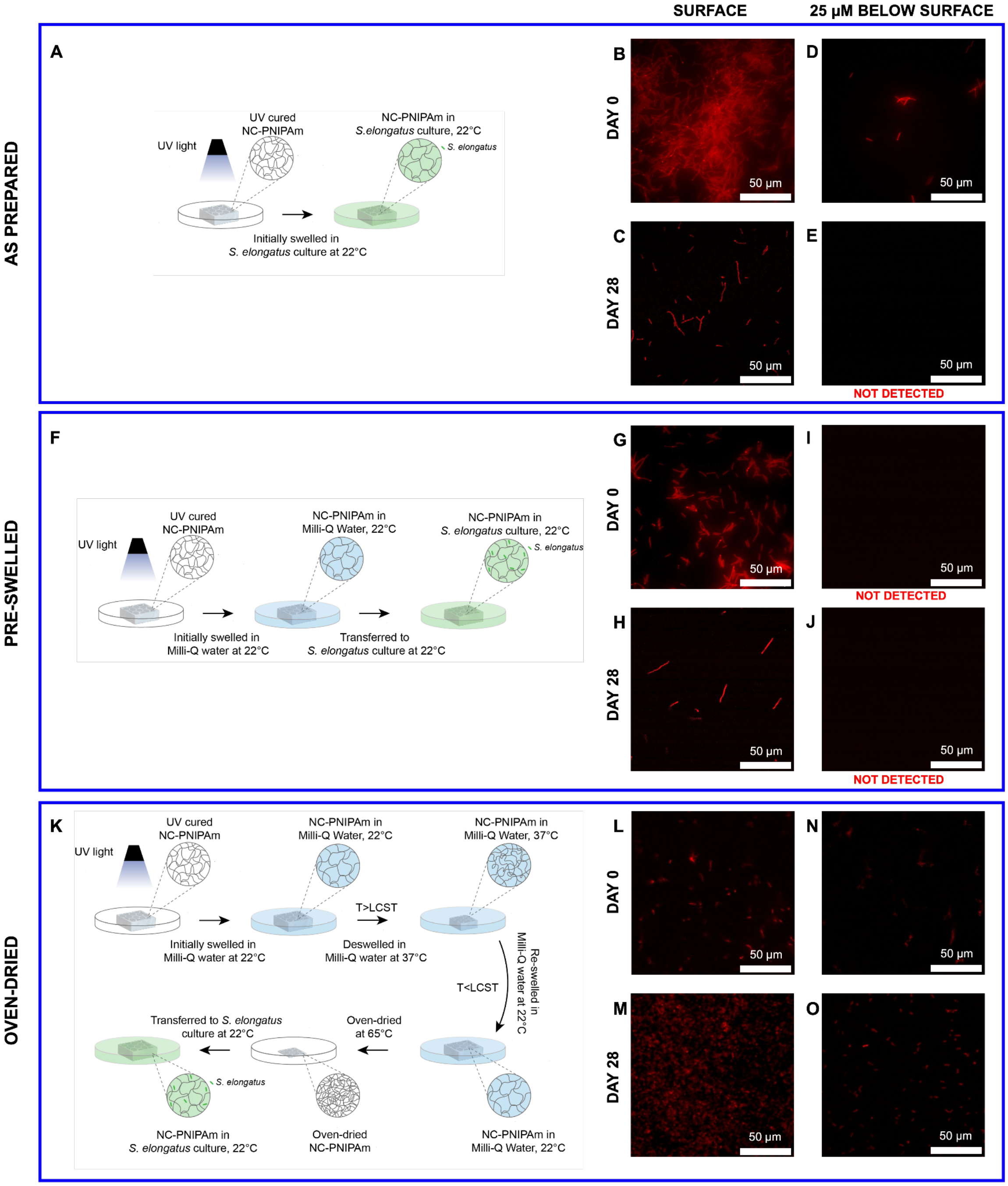

Fig. S2. Alternative diffusion methods. (A) Schematic depicting the first alternative diffusion method, labeled “As Prepared”, where a freshly UV-crosslinked sample of NC-PNIPAm is swelled in a culture of *S. elongatus* cells. **(B-E)** Confocal imaging of chlorophyll *a* autofluorescence showing live cells within NC-PNIPAm/Se prepared using the first alternative method. Images were collected from the surface of the disc on **(B)** Day 0 and **(C)** Day 28, and 25 μm below the surface on **(D)** Day 0 and **(E)** Day 28. **(F)** Schematic depicting the second alternative diffusion method, labeled “Pre-Swelled”, where UV crosslinked NC-PNIPAm is pre-swelled in Milli-Q water at 22°C and then transferred to *S. elongatus* culture. **(G-J)** Confocal images of autofluorescence from live cells within NC-PNIPAm/Se prepared using the second alternative method. Images were collected from the surface of the disc on **(G)** Day 0 and **(H)** Day 28, and 25 μm below the surface on **(I)** Day 0 and **(J)** Day 28. **(K)** Schematic depicting the third alternative diffusion method, labeled “Oven-Dried”, where UV crosslinked NC-PNIPAm is purged of its excess NIPAm monomer by swelling in Milli-Q water,

de-swelling in Milli-Q water, and re-swelling in Milli-Q water, followed by drying in a convection oven at 65°C overnight until fully dried, then rehydrating the dried NC-PNIPAm in liquid *S. elongatus* culture. **(L-O)** Confocal images of autofluorescence from live cells within NC-PNIPAm/Se prepared using the third alternative method. Images were collected from the surface of the disc on **(L)** Day 0 and **(M)** Day 28, and 25 μm below the surface on **(N)** Day 0 and **(O)** Day 28.

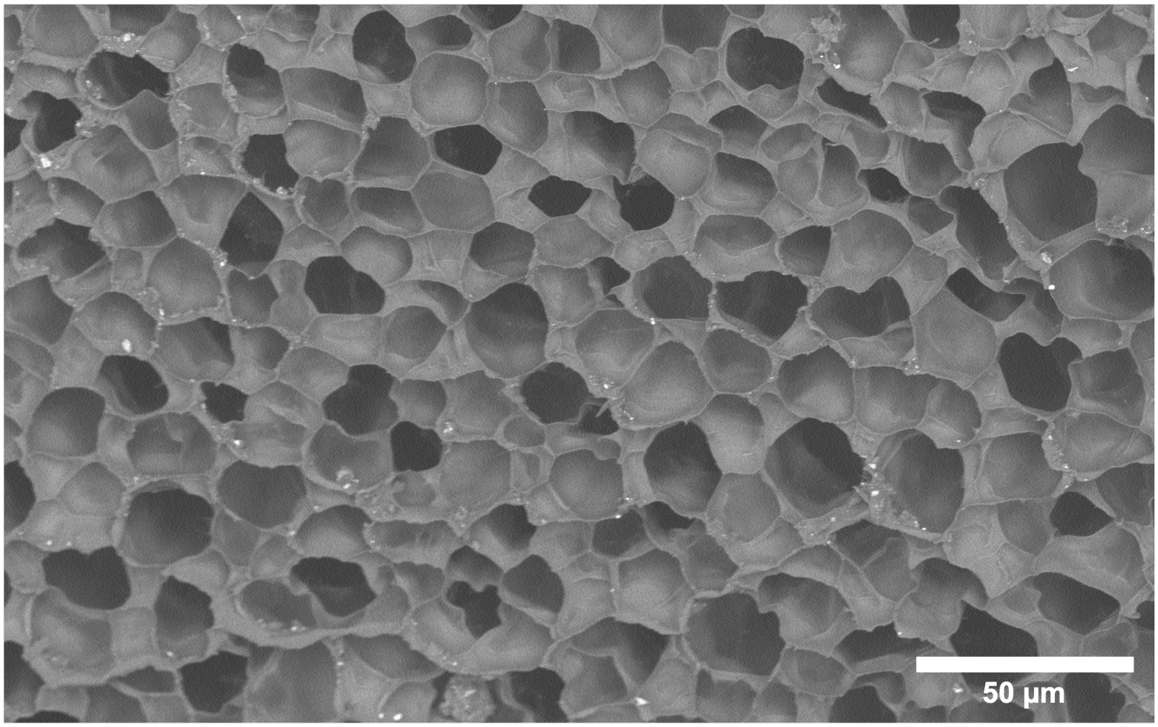

Fig. S3. Scanning electron microscopy. Representative scanning electron microscopy (SEM) image of swelled and subsequently freeze-dried NC-PNIPAm and its average Feret's diameter as measured through thresholding analysis in FIJI, demonstrating an average pore size of 17.8 ± 3.8 µm about nine times larger than the average length of *S. elongatus* cells of approximately 2 µm.

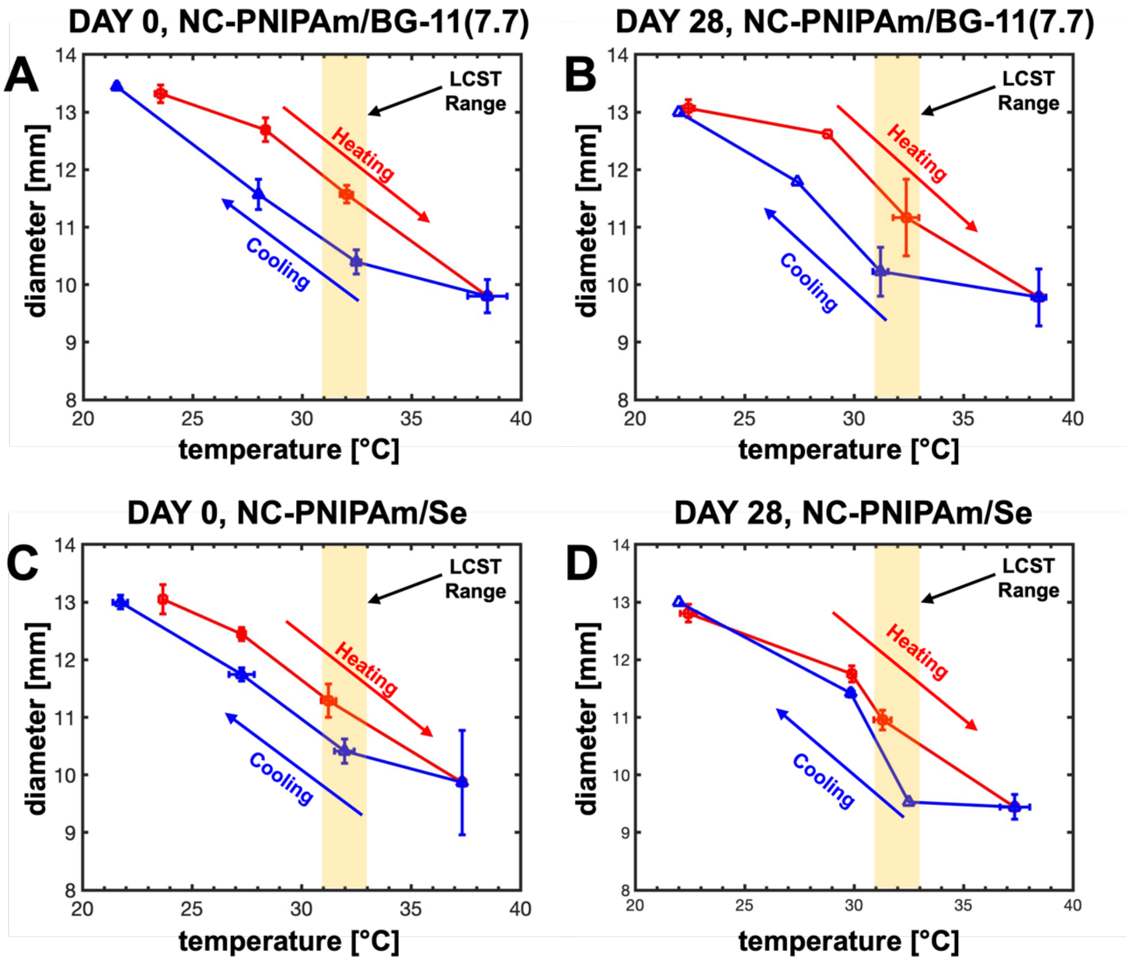

Fig. S4. Testing lower critical solution temperature (LCST) through heating and cooling cycles. The diameters of triplicate discs of NC-PNIPAm/BG-11(7.7) were measured at different temperatures on **(A)** Day 0 and **(B)** Day 28. Triplicate discs of NC-PNIPAm/Se were measured at different temperatures on **(C)** Day 0 and **(D)** Day 28. Heating and cooling curves are indicated in red and blue, respectively. The LCST range between 31-33°C is shown in yellow. Even after 28 days, ELMs NC-PNIPAm/BG-11(7.7) and NC-PNIPAm/Se exhibited similar changes in diameter in response to temperature, indicating that the shape-morphing quality of NC-PNIPAm is not impacted by *S. elongatus* growth.

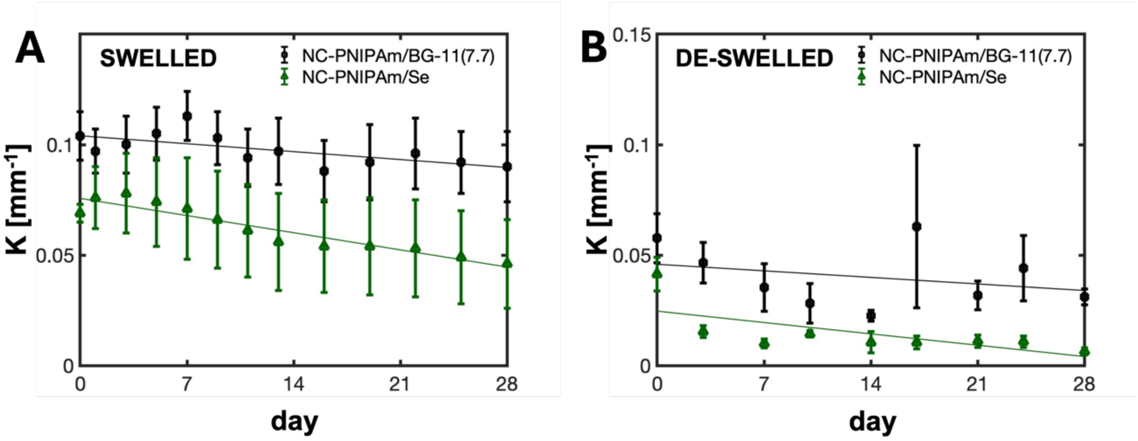

Fig. S5. Temperature-dependent bending curvature changes over 28 days in 100 µm thick NC-PNIPAm/Se compared to 100 µm thick NC-PNIPAm/BG-11(7.7). Bending curvatures were measured in the **(A)** swelled and **(B)** de-swelled states. Data points and error bars for NC-PNIPAm/BG-11(7.7) and NC-PNIPAm/Se are shown in black and green, respectively. Green (r^2=0.89, r^2=0.46) and black (r^2=0.45, r^2=0.09) lines indicate linear regression curves for bending curvature data in swelled and de-swelled states of NC-PNIPAm/Se and NC-PNIPAm/BG-11(7.7), respectively.

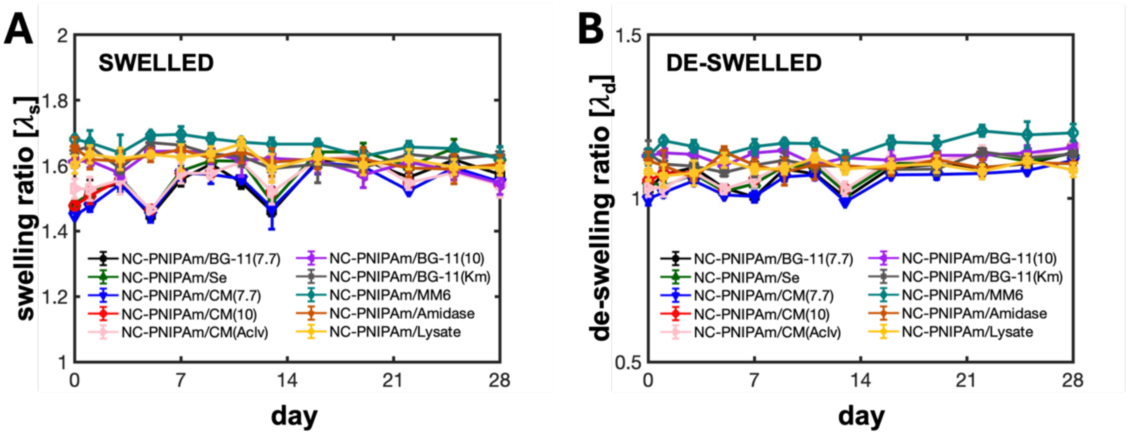

Fig. S6. Changes in the swelling and de-swelling ratios of NC-PNIPAm in ten conditions over 28 days. (A) The linear swelling and **(B)** de-swelling ratios were monitored over a 28-day observation period. Data points, error bars, and lines are shown for ten tested ELMs or hydrogel conditions: NC-PNIPAm/BG-11(7.7) (black), NC-PNIPAm/Se (green), NC-PNIPAm/CM(7.7) (blue), NC-PNIPAm/CM(10) (red), NC-PNIPAm/CM(Aclv) (pink), NC-PNIPAm/BG-11(10) (purple), NC-PNIPAm/BG-11(Km) (gray), NC-PNIPAm/MM6 (teal), NC-PNIPAm/Amidase (orange), and NC-PNIPAm/Lysate (yellow). The observed changes in the linear swelling ratio and linear de-swelling ratio are summarized in **Supporting Table 3**.

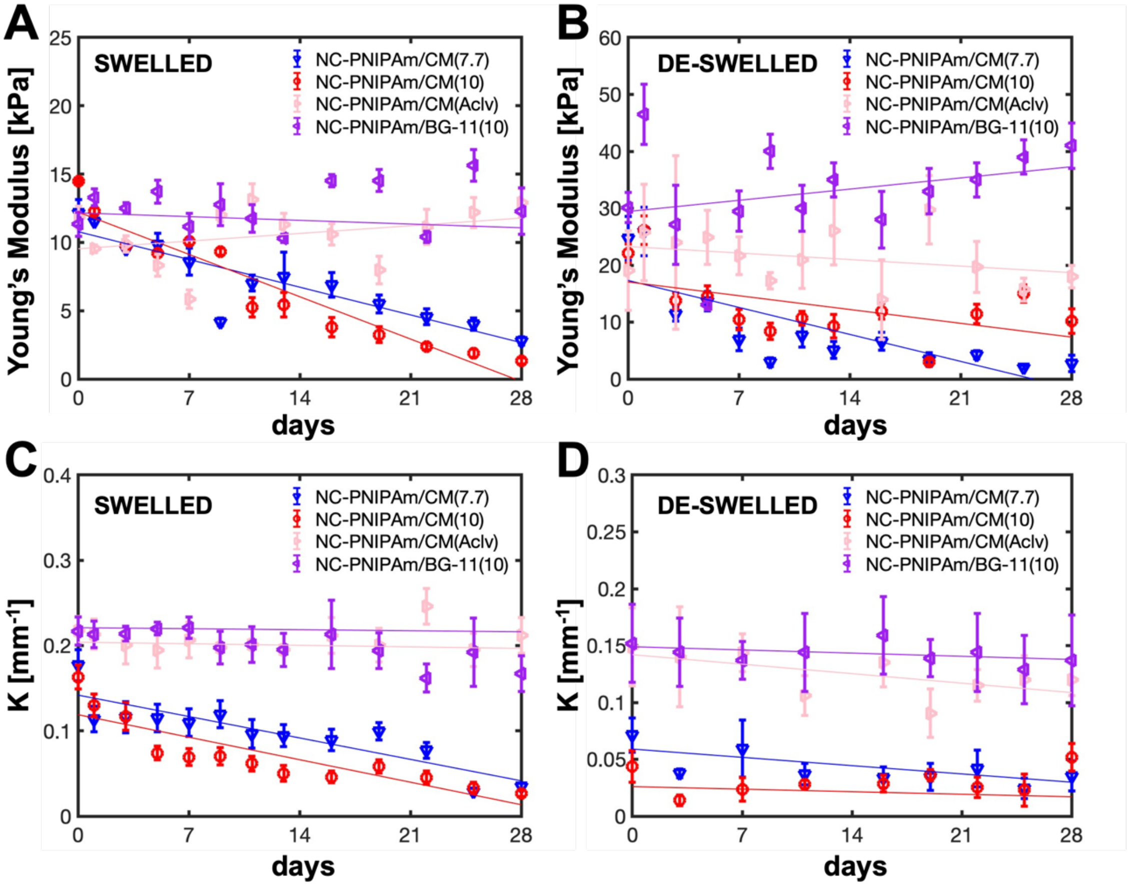

Fig. S7. Changes in local Young's modulus and bending curvature of NC-PNIPAm/CM(7.7), NC-PNIPAm/CM(10), NC-PNIPAm/CM(Aclv), and NC-PNIPAm/BG-11(10) over 28 days. The local Young's modulus at each time point was determined via nanoindentation of each material in the **(A)** swelled and **(B)** de-swelled states. Blue (r^2=0.80, r^2=0.61), red (r^2=0.91, r^2=0.29), pink (r^2=0.12, r^2=0.11), and purple (r^2=0.05, r^2=0.10) lines indicate linear regression curves for the local Young's modulus data in swelled and de-swelled states of NC-PNIPAm/CM(7.7), NC-PNIPAm/CM(10), NC-PNIPAm/CM(Aclv), and NC-PNIPAm/BG-11(10), respectively. The observed changes in the local Young's modulus values are summarized in **Supporting Table 4**. Bending curvatures were measured at each time point in the **(C)** swelled and **(D)** de-swelled states. Blue (r^2=0.78, r^2=0.50), red (r^2=0.75, r^2=0.08), pink (r^2=0.03, r^2=0.37), and purple (r^2=0.67, r^2=0.20) lines indicate linear regression curves for bending curvature data in swelled and de-swelled states of NC-PNIPAm/CM(7.7), NC-PNIPAm/CM(10), NC-PNIPAm/CM(Aclv), and NC-PNIPAm/BG-11(10), respectively. The observed changes in bending curvature values are summarized in **Supporting Table 1**.

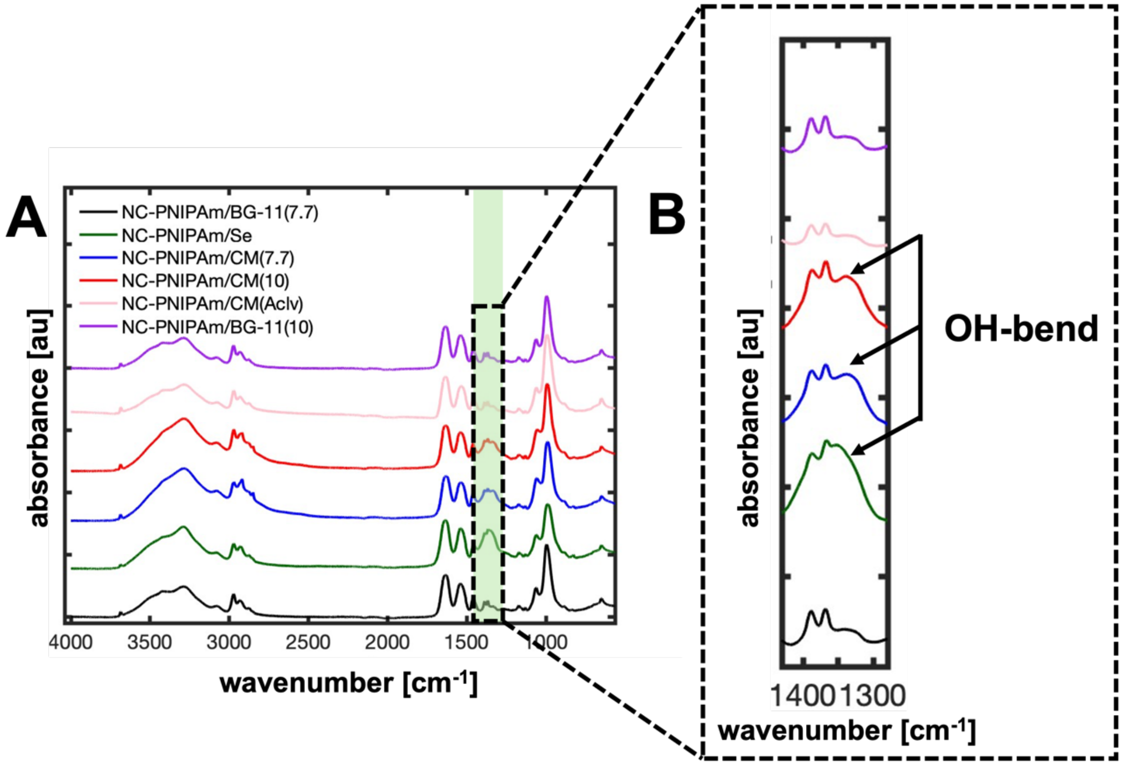

Fig. S8. ATR-FTIR spectra of NC-PNIPAm/BG-11(7.7), NC-PNIPAm/Se, NC-PNIPAm/CM(7.7), NC-PNIPAm/CM(10), NC-PNIPAm/CM(Aclv), and NC-PNIPAm/BG-11(10). Each material was allowed to grow or age for 28 days prior to acquisition of **(A)** ATR-FTIR spectra to elucidate their chemical composition. **(B)** The zoomed-in panel shows the region (1440-1395 wavenumber cm^{-1}) where an OH-bend characteristic of a carboxylic acid, indicated with an arrow, is present for NC-PNIPAm/CM(7.7) (blue), NC-PNIPAm/CM(10) (red), and NC-PNIPAm/Se (green), which are the samples incorporating live cells or their undenatured products.

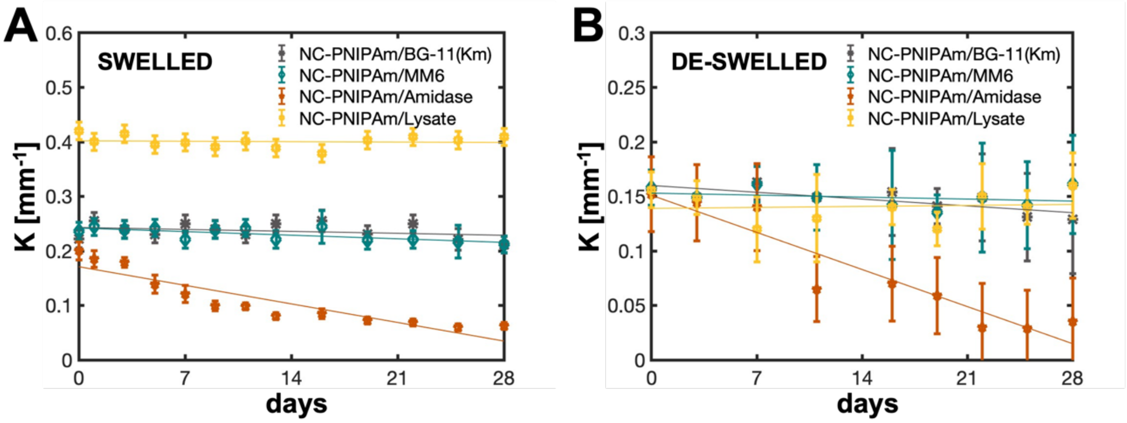

Fig. S9. Monitoring the bending curvature of NC-PNIPAm/BG-11(Km), NC-PNIPAm/MM6, NC-PNIPAm/Amidase, and NC-PNIPAm/Lysate over 28 days. Bending curvatures were measured in the **(A)** swelled and **(B)** de-swelled states of these materials over a 28-day observation period. Gray (r^2=0.11, r^2=0.61), teal (r^2=0.53, r^2=0.08), orange (r^2=0.83, r^2=0.89), and yellow (r^2=0.01, r^2=0.01) lines indicate linear regression curves for the curvature data in both swelled and de-swelled states of NC-PNIPAm/BG-11(Km), NC-PNIPAm/MM6, NC-PNIPAm/Amidase, and NC-PNIPAm/Lysate, respectively. The observed changes in bending curvature values are summarized in **Supporting Table 1**.

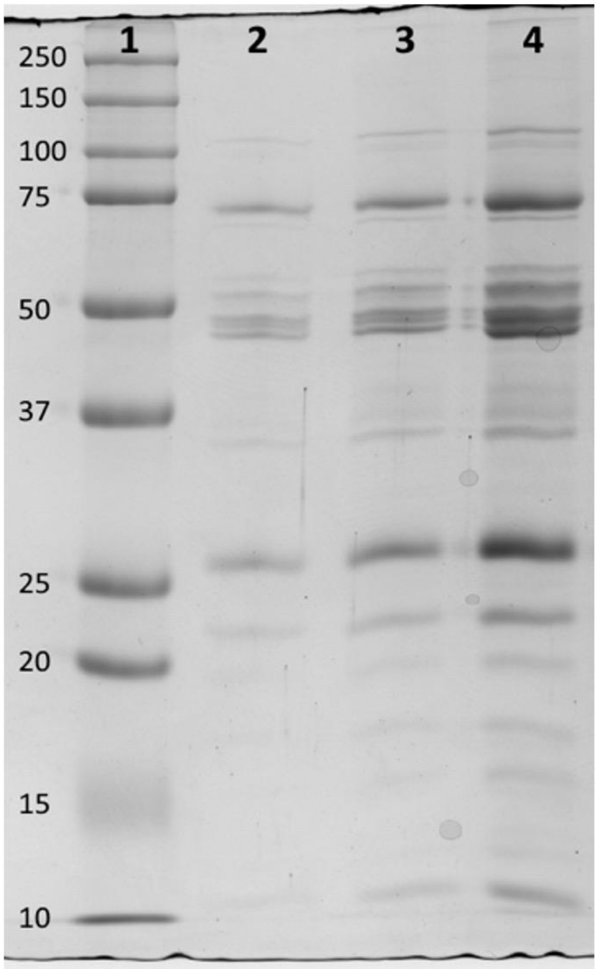

Fig. S10. SDS-PAGE gel visualizing the recombinant putative amidase Synpcc7942_1548 after purification from lysed *E. coli* cells by immobilized metal affinity chromatography. Lane 1 contains a protein ladder with size markers shown in kDa. Lanes 2, 3, and 4 contain 0.5, 1.0, and 5.0 µg of purified, recombinant amidase with a C-terminal hexahistidine tag, respectively. The expected size of the recombinant tagged amidase is 49.2 kDa, or 46.3 kDa without its putative N-terminal signal peptide. The presence of additional, prominent bands suggests co-purification of other proteins or, in the case of smaller bands, possible degradation products of the target protein.

Table S1. Bending curvature of 25 μm thick NC-PNIPAm incorporated with different cells or solutions.

Composition	Swelled Curvature [mm^{-1}]			De-swelled Curvature [mm^{-1}]		
	Day 0	Day 28	Percent Change	Day 0	Day 28	Percent Change
**NC-PNIPAm/Se**	0.167 ± 0.013	0.048 ± 0.007	- 71%	0.028 ± 0.009	0.009 ± 0.002	- 68%
**NC-PNIPAm/BG-11(7.7)**	0.386 ± 0.014	0.465 ± 0.014	+ 20%	0.120 ± 0.063	0.219 ± 0.009	+ 83%
**NC-PNIPAm/CM(7.7)**	0.177 ± 0.018	0.035 ± 0.004	- 80%	0.071 ± 0.015	0.035 ± 0.013	- 51%
**NC-PNIPAm/CM(10)**	0.163 ± 0.014	0.027 ± 0.004	- 83%	0.044 ± 0.014	0.052 ± 0.012	+ 18%
**NC-PNIPAm/CM(AcIv)**	0.217 ± 0.017	0.212 ± 0.021	- 2%	0.149 ± 0.034	0.120 ± 0.014	- 19%
**NC-PNIPAm/BG-11(10)**	0.217 ± 0.016	0.192 ± 0.040	- 12%	0.152 ± 0.034	0.137 ± 0.040	- 10%
**NC-PNIPAm/MM6**	0.236 ± 0.016	0.212 ± 0.015	- 10%	0.158 ± 0.016	0.161 ± 0.045	+ 2%
**NC-PNIPAm/BG-11(Km)**	0.222 ± 0.016	0.211 ± 0.015	- 5%	0.156 ± 0.016	0.129 ± 0.050	- 17%
**NC-PNIPAm/Amidase**	0.200 ± 0.016	0.062 ± 0.006	- 69%	0.152 ± 0.034	0.035 ± 0.040	- 77%
**NC-PNIPAm/Lysate**	0.420 ± 0.016	0.409 ± 0.016	- 3%	0.156 ± 0.016	0.160 ± 0.030	+ 3%

Table S2. Abbreviation definitions and their respective material compositions.

Abbreviation	Composition
**NC-PNIPAm/Se**	NC-PNIPAm diffused with *S. elongatus* cultured in BG-11
**NC-PNIPAm/BG-11(7.7)**	NC-PNIPAm diffused with BG-11 media buffered to pH=7.75
**NC-PNIPAm/CM(7.7)**	NC-PNIPAm diffused with *S. elongatus* conditioned media buffered to pH=7.75
**NC-PNIPAm/CM(10)**	NC-PNIPAm diffused with *S. elongatus* conditioned media buffered to pH=10.00
**NC-PNIPAm/CM(Aclv)**	NC-PNIPAm diffused with *S. elongatus* conditioned media treated with proteinase K and autoclaved
**NC-PNIPAm/BG-11(10)**	NC-PNIPAm diffused with *S. elongatus* BG-11 media buffered to pH=10.00
**NC-PNIPAm/MM6**	NC-PNIPAm diffused with mutant MM6 cultured in BG-11 media with 5 μg/mL kanamycin antibiotic
**NC-PNIPAm/BG-11(Km)**	NC-PNIPAm diffused with BG-11 media with 5 μg/mL kanamycin antibiotic
**NC-PNIPAm/Amidase**	NC-PNIPAm diffused with solution of purified Synpcc7942_1548 protein
**NC-PNIPAm/Lysate**	NC-PNIPAm diffused with clarified lysate from *E. coli* cells expressing Synpcc7942_1548

Table S3. Linear swelling and de-swelling ratios of NC-PNIPAm incorporated with different cells or solutions.

Composition	Linear Swelling Ratio (λ_s)			Linear De-swelling Ratio (λ_d)		
	Day 0	Day 28	Percent Change	Day 0	Day 28	Percent Change
**NC-PNIPAm/Se**	1.485 ± 0.013	1.624 ± 0.018	+ 9%	1.054 ± 0.047	1.137 ± 0.023	+ 8%
**NC-PNIPAm/BG-11(7.7)**	1.471 ± 0.027	1.574 ± 0.022	+ 7%	1.026 ± 0.034	1.141 ± 0.007	+ 11%
**NC-PNIPAm/CM(7.7)**	1.448 ± 0.008	1.551 ± 0.010	+ 7%	0.999 ± 0.020	1.117 ± 0.022	+ 12%
**NC-PNIPAm/CM(10)**	1.477 ± 0.010	1.542 ±0.039	+ 4%	1.054 ± 0.012	1.160 ± 0.018	+ 10%
**NC-PNIPAm/CM(AcIv)**	1.530 ± 0.030	1.542 ± 0.039	+ 1%	1.032 ± 0.025	1.160 ± 0.018	+ 12%
**NC-PNIPAm/BG-11(10)**	1.613 ± 0.018	1.460 ± 0.034	- 9%	1.130 ± 0.011	1.155 ± 0.022	+ 2%
**NC-PNIPAm/MM6**	1.681 ± 0.011	1.621 ± 0.039	- 4%	1.237 ± 0.025	1.201 ± 0.026	- 3%
**NC-PNIPAm/BG-11(Km)**	1.646 ± 0.020	1.632 ± 0.025	- 1%	1.390± 0.038	1.136 ± 0.008	- 18%
**NC-PNIPAm/Amidase**	1.655 ± 0.031	1.604 ± 0.027	- 3%	1.115 ± 0.026	1.106 ± 0.005	- 1%
**NC-PNIPAm/Lysate**	1.604 ± 0.028	1.593 ± 0.023	- 1%	1.080 ± 0.027	1.086 ± 0.021	+ 1%

Table S4. Local Young's modulus of NC-PNIPAm incorporated with different cells or solutions.

Composition	Swelled Local Young's Modulus [kPa]			De-swelled Local Young's Modulus [kPa]		
	Day 0	Day 28	Percent Change	Day 0	Day 28	Percent Change
NC-PNIPAm/Se	13.359 ± 0.773	0.759 ± 0.075	- 94%	18.799 ± 1.150	4.513 ± 1.202	- 76%
NC-PNIPAm/BG-11(7.7)	16.432 ± 0.509	12.420 ± 1.258	-24%	21.466 ± 0.833	22.903 ± 2.576	+ 7%
NC-PNIPAm/CM(7.7)	12.176 ± 0.973	2.780 ± 0.307	- 77%	24.738 ± 3.867	2.715 ± 1.435	- 89%
NC-PNIPAm/CM(10)	14.465 ± 0.114	1.334 ± 0.231	- 91%	22.080 ± 1.680	10.215 ± 2.154	- 54%
NC-PNIPAm/CM(AcIv)	11.841 ± 0.794	12.920 ± 1.012	+ 9%	19.046 ± 6.977	15.714 ± 4.691	- 17%
NC-PNIPAm/BG-11(10)	11.328 ± 0.873	12.294 ± 1.701	+ 9%	30.177 ± 2.634	36.342 ± 4.300	+ 20%
NC-PNIPAm/MM6	6.298 ± 0.773	4.411 ± 0.366	- 30%	14.962 ± 1.003	16.111 ± 2.344	+ 8%
NC-PNIPAm/BG-11(Km)	7.772 ± 0.509	4.616 ± 1.321	- 41%	16.669 ± 1.933	22.669 ± 2.576	+ 36%
NC-PNIPAm/Amidase	8.152 ± 1.169	1.665 ± 0.343	- 80%	17.852 ± 3.246	4.376 ± 0.866	- 75%
NC-PNIPAm/Lysate	10.970 ± 1.079	11.510 ± 0.445	+ 5%	24.153 ± 2.296	26.871 ± 3.860	+ 11%

Table S5. DNA primers used in this study with restriction sites in capital letters.

Primer Name	Primer Sequence (5'-3')
**1548 F**	gtggcaaaacgtcgtgattttttgttagg
**1548 R**	ctaggacacccaagtagatttgggc
**6HAm2991 F**	caggaaacagaccatggGAATTCatgcatcatcatcatcatcatatggcaaaacgtcgtg
**Am2991 R**	gcatgcctgcaggtcgactctagaGGATCCctaggacacccaagtagat
**Am2991 F**	atttcacacaggaaacagaccatgGAATTCatggcaaaacgtcgtg
**Am6H2991 R**	aggtcgactctagaGGATCCttagtgatggtgatggtgatgggacacccaagtagatttg
**pTRC Fwd**	ctgttgacaattaatcatccgg
**2991 CSRev**	cgctacggcgtttcacttctgagttc

Table S6. Plasmids used in this study.

Name	Description	Source
**pAM2991**	Contains the LacI repressor, *pTRC* promoter, *aadA* resistance marker, and flanking regions for homologous recombination into the *S. elongatus* genome (neutral site 1)	*(3)*
**pAmidase6H**	The Synpcc7942_1548 gene with a 3' extension encoding a hexahistidine tag was inserted into the EcoRI site of pAM2991 for heterologous, IPTG-inducible expression in *E. coli*	This study
**p8S1-MM6**	Unigene set plasmid for insertional inactivation of Synpcc7942_1548	*(4, 5)*

Movie S1. *S. elongatus* diffusion into NC-PNIPAm. Time lapsed video of *S. elongatus* cell diffusion into thin NC-PNIPAm sheet (20 mm x 5 mm x 0.25 mm). Video is comprised of sequential images captured every 5 seconds over 5 minutes using fluorescence microscopy. Time lapse images were stitched together are played at 25X speed.

References:

1. N. B. Ivleva, M. R. Bramlett, P. A. Lindahl, S. S. Golden, LdpA: a component of the circadian clock senses redox state of the cell. *The EMBO Journal* **24**, 1202-1210 (2005).
2. D. G. Gibson *et al.*, Enzymatic assembly of DNA molecules up to several hundred kilobases. *Nature Methods* **6**, 343-345 (2009).
3. C. K. Holtman *et al.*, High-Throughput Functional Analysis of the Synechococcus elongatus PCC 7942 Genome. *DNA Research* **12**, 103-115 (2005).
4. M. Adomako *et al.*, Comparative Genomics of Synechococcus elongatus Explains the Phenotypic Diversity of the Strains. *mBio* **13**, e00862-00822 (2022).
5. Y. Chen, C. K. Holtman, A. Taton, S. S. Golden, "Functional Analysis of the Synechococcus elongatus PCC 7942 Genome" in Functional Genomics and Evolution of Photosynthetic Systems*,* R. Burnap, W. Vermaas, Eds. (Springer Netherlands, Dordrecht, 2012), pp. 119-137.
